# Bone-Targeting AAV-Mediated Gene Silencing in Osteoclasts for Osteoporosis Therapy

**DOI:** 10.1016/j.omtm.2020.04.010

**Published:** 2020-04-18

**Authors:** Yeon-Suk Yang, Jun Xie, Sachin Chaugule, Dan Wang, Jung-Min Kim, JiHea Kim, Phillip W.L. Tai, Seok-kyo Seo, Ellen Gravallese, Guangping Gao, Jae-Hyuck Shim

**Affiliations:** 1 Division of Rheumatology, University of Massachusetts Medical School, Worcester, MA, USA; 2 Horae Gene Therapy Center, University of Massachusetts Medical School, Worcester, MA, USA; 3 Department of Microbiology and Physiological Systems, University of Massachusetts Medical School, Worcester, MA, USA; 4 Viral Vector Core, University of Massachusetts Medical School, Worcester, MA, USA; 5 Department of Obstetrics and Gynecology, Severance Hospital, Yonsei University College of Medicine, Seoul, Korea; 6 Division of Rheumatology, Immunology and Allergy, Brigham and Women’s Hospital, Boston, MA, USA; 7 Li Weibo Institute for Rare Diseases Research, University of Massachusetts Medical School, Worcester, MA, USA

**Keywords:** osteoporosis, osteoclast, osteoblast, rAAV, artificial miRNA, cathepsin k, rank, bone resorption, bone formation, osteoclastogenesis

## Abstract

Improper activity of bone-resorbing osteoclasts results in low bone density and deterioration of bone structure, which increase the risk of fractures. Anti-resorptive therapies targeting osteoclasts have proven effective in preserving bone mass, but these therapeutic agents lead to defective new bone formation and numerous potential side effects. In this study, we demonstrate that recombinant adeno-associated virus, serotype 9 (rAAV9) can deliver to osteoclasts an artificial microRNA (amiR) that silences expression of key osteoclast regulators, RANK (receptor activator for nuclear factor κB) and cathepsin K (rAAV9.*amiR-rank*, rAAV9.*amiR-ctsk*), to prevent bone loss in osteoporosis. As rAAV9 is highly effective for the transduction of osteoclasts, systemic administration of rAAV9 carrying *amiR-rank* or *amiR-ctsk* results in a significant increase of bone mass in mice. Furthermore, the bone-targeting peptide motif (Asp)_14_ or (AspSerSer)_6_ was grafted onto the AAV9-VP2 capsid protein, resulting in significant reduction of transgene expression in non-bone peripheral organs. Finally, systemic delivery of bone-targeting rAAV9.*amiR-ctsk* counteracts bone loss and improves bone mechanical properties in mouse models of postmenopausal and senile osteoporosis. Collectively, inhibition of osteoclast-mediated bone resorption via bone-targeting rAAV9-mediated silencing of *ctsk* is a promising gene therapy that can preserve bone formation and mitigate osteoporosis, while limiting adverse off-target effects.

## Graphical Abstract

**Figure:**
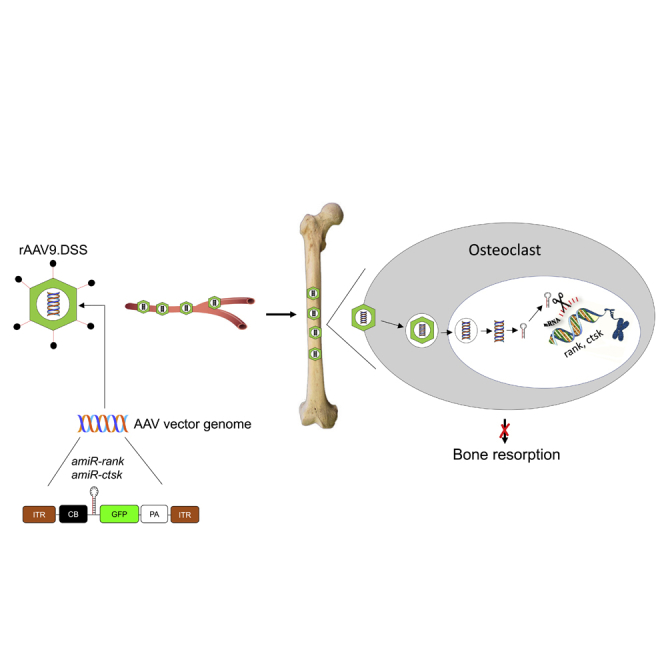

## Teaser

Anti-resorptive therapies targeting osteoclasts have proven effective in preserving bone mass, but these therapeutic agents lead to numerous side effects. The authors show that silencing cathepsin K in osteoclasts by delivering an artificial miRNA via bone-homing AAV9 can counteract bone loss in mouse models of postmenopausal and senile osteoporosis.

## Introduction

Osteoporosis is an aging-associated disease marked by bone loss and deterioration of the bone microstructure.^1^ Collectively, these factors significantly increase the risk of fractures. Most existing therapeutic agents for osteoporosis are anti-resorptive agents, such as bisphosphonates and anti-RANKL (receptor activator of nuclear factor κB ligand) antibody, inhibitors of osteoclast (OC) differentiation and bone resorption. Unfortunately, their effectiveness is limited by an inability to promote new bone formation, as OC-mediated bone resorption is coupled with osteoblast-mediated bone formation during remodeling.^2^^,^^3^ Moreover, these agents are accompanied by potential adverse effects, including atypical fractures and osteonecrosis of the jaw.^4^^,^^5^ Thus, the development of anti-resorptive therapies that can preserve osteoblast function while limiting off-target side effects is still an unmet need.

OCs originate from hematopoietic stem cells (HSCs) in the bone marrow and require macrophage-colony stimulating factor (M-CSF) and RANKL for the differentiation of monocytes to multinucleated OCs. The loss of M-CSF and its receptor c-Fms,^6^ or RANKL and its receptor RANK,^7^^,^^8^ results in severe osteopetrosis and abnormal tooth eruption in mice because of a complete absence of mature OCs. Osteoprotegerin (OPG), a soluble decoy receptor for RANKL, acts as a natural suppressor of RANKL-RANK signaling by obstructing RANKL binding to RANK on the surface of OC precursor cells.^9^ Similarly, the human monoclonal immunoglobulin G (IgG)2 antibody against RANKL (denosumab) prevents RANKL from activating RANK, thereby inhibiting OC survival and differentiation.^10^ Despite its favorable therapeutic efficacy and safety profile, continuous treatment with denosumab reduces physiologic bone remodeling and causes potential adverse effects.^3^

Cathepsin K (CTSK), a cysteine protease member of the cathepsin lysosomal protease family, is highly expressed in OCs and efficiently degrades type 1 collagen, the major component of organic bone matrix.^11^ Mice lacking CTSK display osteopetrosis as a consequence of impaired bone resorption, while bone formation is normal or increased.^12^^,^^13^ In human clinical trials, the pharmacologic CTSK inhibitor odanacatib showed a continuous increase in bone mineral density and enhanced bone strength at the hip and spine. However, as CTSK is expressed in various nonskeletal tissues, including skin, cardiovascular, and cerebrovascular sites, its pharmacological inhibition causes an increased risk of off-target cerebrovascular accidents.^14^, ^15^, ^16^

Adeno-associated virus (AAV), a small non-enveloped parvovirus with a single-stranded genome, has been used for gene therapy in more 140 clinical trials, involving more than 2,000 patients worldwide. Recombinant AAVs (rAAVs) show high transduction efficiency, persistent transgene expression, and generally lack post-infection immunogenicity and pathogenicity.^17^, ^18^, ^19^ The AAV vector genome contains two inverted terminal repeats (ITRs) and regulatory (*rep*) and structural capsid (*cap*) open reading frames (ORFs). A transgene of interest can replace the *rep* and *cap* ORFs, producing a replication-defective rAAV genome.^20^^,^^21^ Additionally, self-complementary AAV (scAAV) vectors were developed to enhance *in vitro* and *in vivo* transduction efficacies.^22^, ^23^, ^24^, ^25^ Using a bone-targeting AAV9 capsid, this study aimed to develop an osteoporosis therapy that simultaneously suppresses OC-mediated bone resorption and promotes osteoblast-mediated bone formation, while limiting off-target side effects. We provide a proof-of-concept demonstration that a rationally designed AAV9 capsid can deliver an artificial microRNA (amiR) that silences the expression of RANK (OC survival/differentiation) or CTSK (OC resorption activity) in OCs to reverse bone loss and improve bone mechanical properties in mouse models of postmenopausal and senile osteoporosis, while detargeting transduction to non-relevant tissues.

## Results

### rAAV9 Can Effectively Transduce OCs

**Figure 1:**
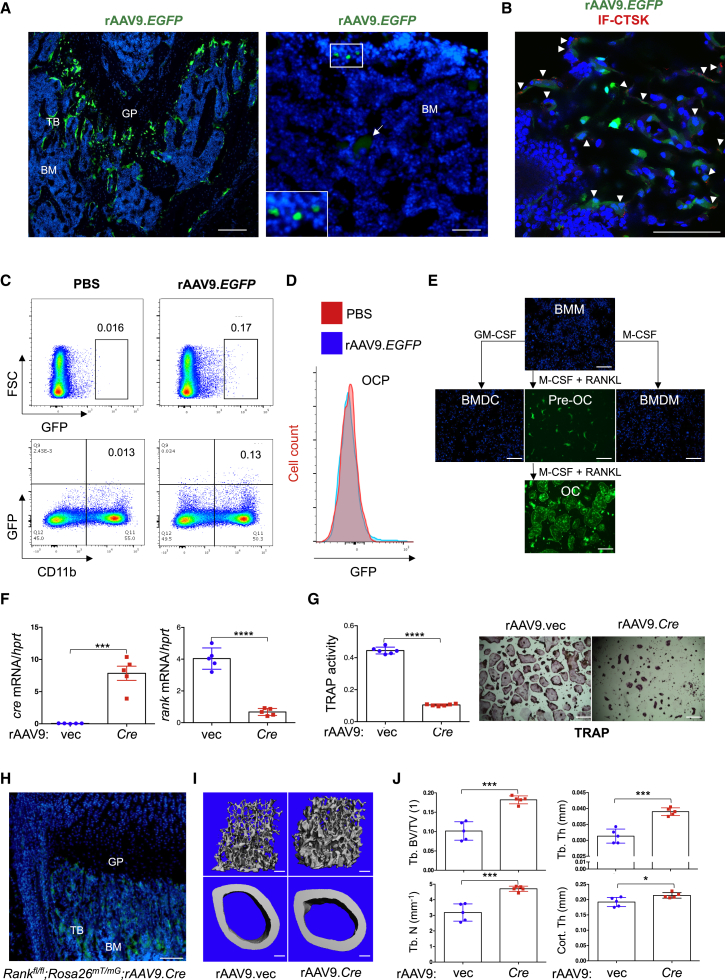
rAAV9 Transduces Osteoclast Lineage Cells *In Vitro* and *In Vivo* (A–D) A single dose of PBS or 8 × 10^11^ genome copies (GC) of rAAV.*EGFP* was intravenously (i.v.) injected into 2-month-old male mice, and EGFP expression was assessed in cryosectioned femurs by fluorescence microscopy 2 weeks post-injection. (A) Arrow indicates megakaryocytes with autofluorescence. TB, trabecular bone; BM, bone marrow; GP, growth plate (n = 3/group). Scale bars, 100 μm. (B) Cells were also immunostained with anti-CTSK antibody to identify osteoclast (OC)-lineage cells. Arrowheads indicate AAV9-transduced CTSK^+^ OCs. Scale bar, 75 μm. (C and D) Alternatively, EGFP expression in bone marrow cells was assessed by flow cytometry. Flow cytometry gating strategy of OC precursors (OCPs; CD3ε^−^, B220^−^, TER119^−^, CD11b^−/lo^, Ly6c^+^) is described in Figure S2C. GFP-expressing, CD11b-positive cells (C) and OCPs (D) are displayed in the dot plot and histogram, respectively. (E) Bone marrow-derived monocytes (BMMs) were cultured with M-CSF or GM-CSF (granulocyte-macrophage colony-stimulating factor) for 6 days to differentiate into bone marrow-derived macrophages (BMDMs) or dendritic cells (BMDCs), respectively. 10^11^ GC of rAAV9.*EGFP* were used to treat BMMs at day 0, or BMDMs and BMDCs at day 6, of culturing. Transduction efficiencies were assessed by EGFP expression using fluorescence microscopy. Cell nuclei were stained by DAPI. Scale bars, 1 mm. Alternatively, BMMs were cultured with M-CSF and RANKL for 2 and 6 days to differentiate into pre-OCs and mature OCs, respectively. rAAV9.*EGFP* was used to treat pre-OCs at day 2 or mature OCs at day 6 of culturing. (F and G) Two days after treatment with M-CSF and RANKL, *Rank*^*fl/fl*^ pre-OCs were transduced with either rAAV9 carrying *EGFP* control (rAAV9.*vec*) or Cre recombinase (rAAV9.*Cre*) and then differentiated into mature OCs. Levels of (F) *cre* or *rank* mRNA and (G) TRAP activity were measured by RT-PCR (F) and colorimetric assay (G, left). Representative images of TRAP-stained OCs are displayed (G, right). Scale bars, 1 mm. (H–J) A single dose of 8 × 10^11^ GC of rAAV9.*vec* or rAAV9.*cre* was i.v. injected into 3-month-old female *Rank*^*fl/fl*^*;Rosa*^*mTmG*^ mice. Fluorescence microscopy was performed on cryosectioned femurs to identify EGFP-expressing cells 2 weeks post-injection (H), and femoral trabecular bone mass was assessed by microCT 2 months post-injection. Representative 3D reconstruction (I) and relative quantification (J) are displayed. Trabecular bone volume/total volume (Tb.BV/TV), trabecular thickness (Tb.Th), trabecular number per cubic millimeter (Tb.N), and cortical thickness (Cort.Th) are shown (n = 5/group). Scale bars, 200 μm. Values represent mean ± SD. ∗p < 0.05, ∗∗∗p < 0.001 ∗∗∗∗p < 0.0001 by an unpaired two-tailed Student’s t test.

Our previous study demonstrated that systemic delivery of rAAV9 in mice can transduce osteoblast-lineage cells and OCs residing on the bone surface (BS).^26^ Since OCs originate from HSCs in the bone marrow, we explored the ability of rAAV9 to transduce other HSC-lineage cells. rAAV9.*EGFP* was intravenously (i.v.) injected into 2-month-old mice and the tissue distribution of rAAV9 was assessed by EGFP expression using IVIS (*in vivo* imaging system) optical imaging (Figure S1). Individual organ imaging of treated mice showed EGFP expression in the heart, liver, and hindlimbs. In the femur, most EGFP-expressing cells, including osteoblasts and CTSK-expressing OCs, were located in the trabecular bone of the metaphysis, while only a few round-shaped bone marrow cells exhibited EGFP expression (Figures 1A and 1B). Flow cytometry analysis of bone marrow cells confirms EGFP expression in a small subset of CD11b^+^ monocytes, OC progenitors (OCPs; CD3ε^−^, B220^−^, TER119^−^, CD11b^−/lo^, Ly6c^+^), and B220^+^ B lymphocytes (Figures 1C and 1D; Figures S2B and S2C). Of note, megakaryocytes residing in the bone marrow display autofluorescence (Figure 1A, right; Figure S2A). An *in vitro* differentiation assay of bone marrow-derived monocytes (BMMs) revealed that rAAV9 is highly effective for transducing RANKL-treated pre-OCs and mature OCs, but not BMMs, bone marrow-derived macrophages (BMDMs), and bone marrow-derived dendritic cells (BMDCs) (Figure 1E). Thus, these results demonstrate that rAAV9 is more effective in transducing pre-OCs and mature OCs than other HSC-lineage cells in the bone marrow.

RANK is a member of tumor necrosis factor receptor (TNFR) family essential for the differentiation of monocytes to mature OCs.^27^ Accordingly, conditional deletion of a floxed *rank* allele with Cre deleter mice targeting OCs (*Rank*^*fl/fl*^*;Ctsk*) results in osteopetrosis, because of a lack of OC-mediated bone resorption (Figures S3A and S3B). To provide a proof-of-concept demonstration that systemically delivered rAAV9 can target *Rank* in OCs to inhibit bone resorption, we generated an rAAV9 vector expressing Cre recombinase (rAAV9.*Cre*, Figure S3C), which mediates the deletion of *Rank* in OC lineage cells. rAAV9-mediated Cre expression in cultured *Rank*^*fl/fl*^ pre-OCs was effective in deleting *Rank* and ablating OC differentiation (Figures 1E and 1F). To visualize rAAV9-mediated Cre expression in OCs *in vivo*, the Cre-reporter *Rosa*^*mT/mG*^ mice were crossed with *Rank*^*fl/fl*^ mice (*Rank*^*fl/fl*^*;Rosa*^*mT/mG*^). rAAV9.*Cre* was i.v. injected into 2-month-old *Rank*^*fl/fl*^*;Rosa*^*mT/mG*^ mice, and Cre-mediated GFP expression in the femur was validated by fluorescence microscopy 2 weeks post-injection (Figure 1G). Two months after the injection, rAAV9.*Cre*-treated femurs showed a significant increase in trabecular bone mass and cortical thickness relative to rAAV9.*EGFP*-treated femurs (Figures 1H and 1I). These results demonstrate that systemically delivered rAAV9.*Cre* in *Rank*^*fl/fl*^*;Rosa*^*mT/mG*^ mice targets OCs and directs the deletion of *Rank* to increase bone mass.

### rAAV9-Mediated Gene Silencing in OCs Increases Bone Mass in Mice

**Figure 2:**
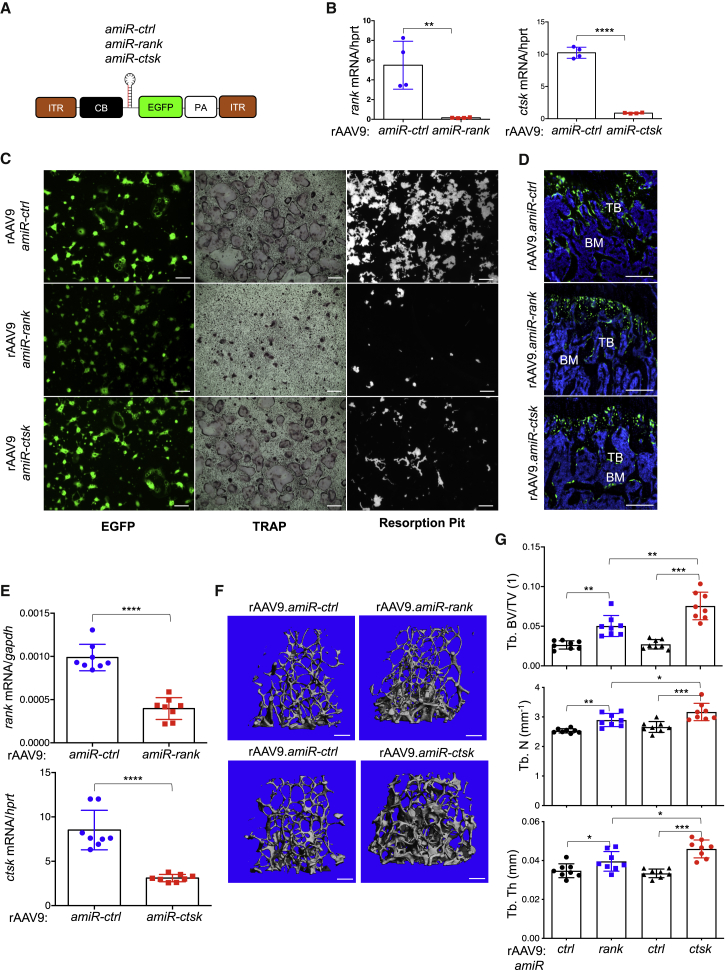
rAAV9-Mediated Gene Silencing in OCs Increases Bone Mass in Mice (A) Diagram of the rAAV9 constructs containing a CMV (cytomegalovirus) enhancer/chicken β-actin promoter (*CB*), *amiR-ctrl*, *amiR-rank*, or *amiR-ctsk*, an *EGFP* reporter gene, *β-globin* poly(A) sequence (PA), and inverted terminal repeats (ITRs). (B and C) Two days after treatment with M-CSF and RANKL, wild-type pre-OCs were transduced with either rAAV9 carrying *amiR-ctrl*, *amiR-rank*, or *amiR-ctsk* (10^11^ GC) and then differentiated into mature OCs. (B) Levels of *rank* or *ctsk* mRNA were measured by RT-PCR and normalized to *hprt*. (C) Transduction efficiency, OC differentiation, and resorption activity were assessed by EGFP expression, TRAP staining, and resorption pit assay, respectively. Representative images of TRAP-stained OCs and resorption pit are displayed (n = 4/group). Scale bars, 1 mm. (D–G) A single dose of 8 × 10^11^ GC of rAAV9 carrying *amiR-ctrl*, *amiR-rank*, or *amiR-ctsk* was i.v. injected into 2-month-old female mice. Two months later, EGFP expression in the cryosectioned femur and mRNA levels of *rank* or *ctsk* in the tibia were assessed by fluorescence microscopy (D) and RT-PCR (E), respectively (n = 8/group). Femoral trabecular bone mass was assessed by microCT. Representative 3D-reconstruction (F) and relative quantification (G) are displayed (n = 8/group). Scale bars, 200 μm. Values represent mean ± SD. *p < 0.05, **p < 0.01, ****p < 0.0001 by an unpaired two-tailed Student’s t test and one-way ANOVA test.

To inhibit OC-mediated bone resorption, we aimed to silence key OC regulators, RANK (*tnfrsf11a*) and CTSK (*ctsk*), using rAAV9-mediated gene transfer. Embedding the guide strand of a small silencing RNA into the miR-33-derived miRNA scaffold (amiR) limits short hairpin RNA (shRNA)-related toxicity, enables efficient gene knockdown, and reduces off-target silencing by 10-fold compared to conventional shRNA constructs.^28^ We therefore generated amiR cassettes targeting two different positions of *rank* (*amiR-rank*) or *ctsk* (*amiR-ctsk*) mRNA and then packaged them with AAV9 capsids (Figure 2A). Of note, the amiR cassette is embedded within the *EGFP* intronic region of the transgene cassette to track transduced cells or tissues. The knockdown efficiency of these vectors in cultured wild-type pre-OCs was examined using RT-PCR (Figure 2B; Figures S4A–S4D). We found that the levels of *rank* or *ctsk* mRNA were more efficiently reduced in the cells when treated with rAAV9.*amiR-rank-2* or *amiR-ctsk-1* than rAAV9.*amiR-rank-1* or *amiR-ctsk-2* (Figures S4B and 4SD). Hereafter and in Figure 2, *amiR-rank-2* and *amiR-ctsk-1* are referred to as *amiR-rank* and *amiR-ctsk*, respectively.

**Figure 3:**
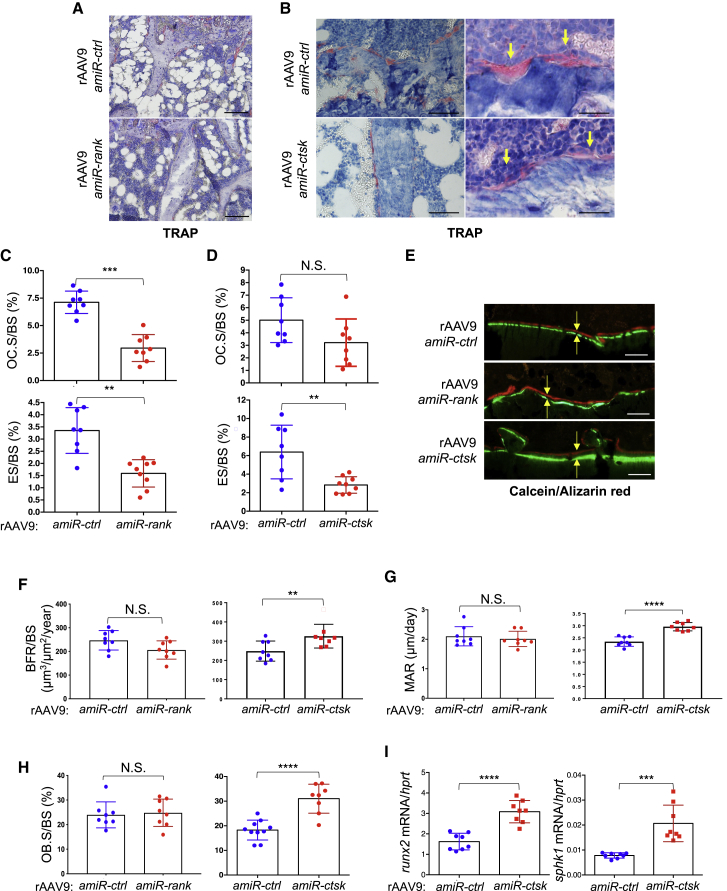
rAAV9-Mediated Gene Silencing in OCs Inhibits Bone Resorption in Mice A single dose of 8 × 10^11^ GC of rAAV9 carrying *amiR-ctrl*, *amiR-rank*, or *amiR-ctsk* was i.v. injected into 2-month-old female mice. Two months later, histomorphometry was performed in the metaphysis of treated femurs to assess *in vivo* OC and osteoblast activities. (A–H) Quantification of OC.S/BS, ES/BS (C and D), BFR/BS, MAR (F and G), OB.S/BS (H), and representative TRAP-stained femurs (A and B) and calcein/alizarin red-labeled femurs (E) are displayed (n = 8/group). Arrows indicate OCs residing on the bone surface (B, right) and the distance between calcein and alizarin red labeling (E). (I) mRNA levels of *runx2* or *sphk1* in the tibia were assessed by RT-PCR. OC.S/BS, osteoclast surface/bone surface. ES/BS, erosion surface/bone surface; BFR/BS, bone formation rate/bone surface; MAR, mineral apposition rate; OB.S/BS, osteoblast surface/bone surface. Scale bars, A and B (left), 100 μm; B (right) and E, 50 μm. Values represent mean ± SD. ∗p < 0.05, ∗∗p < 0.01, ∗∗∗p < 0.001, ∗∗∗∗p < 0.0001 by an unpaired two-tailed Student’s t test and one-way ANOVA test. N.S., not significant.

Genetic deletion of *rank* in mice results in a complete absence of OCs due to defective OC survival and differentiation.^7^^,^^8^ In contrast, loss of CTSK impairs OC resorption activity while preserving OC survival and differentiation.^12^^,^^13^ Similar to the genetic deletion of *rank* or *ctsk*, rAAV9-mediated silencing of *rank* impairs both OC differentiation and resorption activity, whereas only bone resorption activity was markedly reduced in rAAV9.*amiR-ctsk*-treated cells (Figure 2C). To examine the ability of *amiR-rank* or *amiR-ctsk* to inhibit bone resorption *in vivo*, rAAV9 vectors carrying either transgenes were i.v. injected into 2-month-old mice, and two months later, whole-body and femur EGFP expression was visualized by IVIS imaging (Figure S4E) and fluorescence microscopy (Figure 2D), respectively. Compared to rAAV9.*amiR-ctrl*-treated femurs, rAAV9.*amiR-rank- or amiR-ctsk*-treated femurs displayed ~60% reduction of *rank* or *ctsk* mRNA levels and a relative increase in trabecular bone mass (Figures 2F and 2G). Intriguingly, rAAV9.*amiR-ctsk* demonstrates a higher potency of bone accrual than does rAAV9.*amiR-rank*, as shown by the greater trabecular bone volume (BV), number, and thickness in rAAV9.*amiR-ctsk*-treated femurs relative to rAAV9.*amiR-rank-*treated femurs. To gain insight into the mechanism of these findings, histomorphometry was performed in the metaphysis of treated femurs to assess *in vivo* OC and osteoblast activities. In rAAV9.*amiR-rank-*treated femurs, the numbers of tartrate-resistant acid phosphatase (TRAP)-positive OCs and bone erosion surfaces were markedly reduced, compared to rAAV9.*amiR-ctrl*-treated femurs (Figures 3A and 3C). However, treatment with rAAV9.*amiR-ctsk* results in a significant reduction in bone erosion surfaces where flat-shaped OCs reside, while OC numbers were not significantly altered (Figures 3B and 3D). Unlike rAAV9.*amiR-rank-*treated femurs, bone formation rate (BFR), mineral apposition rate (MAR), and osteoblast surface per BS (Ob.S/BS) were markedly increased in rAAV9.*amiR-ctsk*-treated femurs (Figures 3F–3H). These findings demonstrate that treatment with rAAV9.*amiR-rank* results in a significant decrease in OC differentiation and resorption activity without any alteration in osteoblast activity. Alternatively, treatment with rAAV9.*amiR-ctsk* simultaneously impairs OC-mediated bone resorption and promotes osteoblast-mediated bone formation, without affecting OC differentiation. Notably, treatment of cultured osteoblasts with rAAV9.*amiR-ctsk* did not affect osteoblast proliferation and differentiation (Figure S5), demonstrating that increased osteoblast activity seen in rAAV9.*amiR-ctsk*-treated femurs is an indirect effect of rAAV9-mediated silencing of *ctsk*. Instead, similar to mice a with genetic deletion of *ctsk* in OCs,^29^ rAAV9.*amiR-ctsk*-treated femurs display elevated levels of sphingosine kinase 1 (*sphk1*) and runt related transcription factor 2 (*runx2*) mRNA (Figure 3I). Given that SPHK1 promotes osteogenesis by phosphorylating sphingosine to generate sphingosine 1 phosphate (S1P),^30^ rAAV9-mediated silencing of *ctsk* in OCs likely enhances osteoblast-mediated bone formation via increased production of S1P. Taken together, the rAAV9-*amiR-ctsk* vector is a more potent agent for osteoporosis therapy than the rAAV9-*amiR-rank* vector, since *amiR-ctsk* can suppress OC-mediated bone resorption and promote osteoblast-mediated bone formation, simultaneously.

### Development of a rAAV9 Capsid Targeting OCs in the Bone Tissue

**Figure 4:**
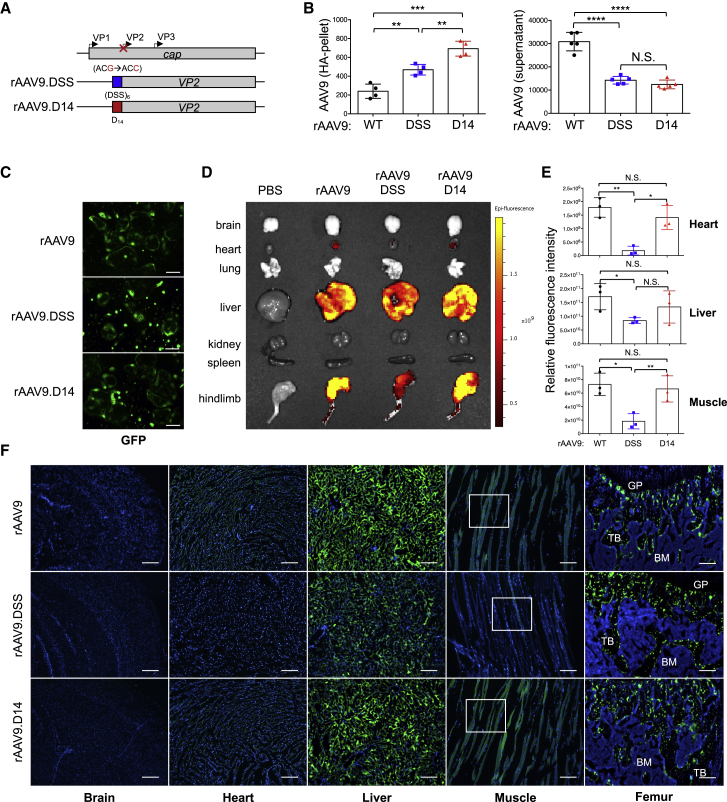
Development of a Bone-Homing rAAaV9 Capsid (A) Diagram of constructs for the rationally designed bone-homing rAAV9 capsids. The bone-targeting-peptide motifs ((DSS)_6_, blue; D_14_, red) were inserted into the AAV9 capsid at the N terminus of AAV9-VP2 (AAV9.DSS-Nter, AAV9.D14-Nter). cap, capsid proteins. (B) Hydroxyapatite (HA)-binding assay. Vectors (10^9^ GC) were incubated with HA beads for 1 h at 37°C and pelleted by centrifugation at 300 rpm. Vector titers in the pellet and supernatant were measured by ddPCR and normalized to PBS control. (C) Two days after treatment with M-CSF and RANKL, wild-type pre-OCs were transduced with vectors (10^11^ GC) and then differentiated into mature OCs. Transduction efficiency was assessed by EGFP expression using fluorescence microscopy. Scale bars, 1 mm. (D–F) A single dose of 8 × 10^11^ GC of vectors was i.v. injected into 2-month-old female mice, and 2 weeks later, EGFP expression in individual tissues was assessed by IVIS 100 optical imaging. (D and E) Representative tissues (D) and relative quantification (E) are displayed (n = 3/group). Scale bars represent relative fluorescence (p/s/cm^2^/sr/μW/cm^2^). (F) Alternatively, EGFP expression was assessed by fluorescence microscopy in cryosectioned brain, heart, liver, skeletal muscle, and femur. Boxes indicate areas of high magnification images displayed in Figure S6B. Scale bars, 100 μm. Values represent mean ± SD. ∗p < 0.05, ∗∗p < 0.01, ∗∗∗p < 0.001, ∗∗∗∗p < 0.0001 by an unpaired two-tailed Student’s t test and one-way ANOVA test. N.S., not significant.

Expression of CTSK is also seen in various non-skeletal tissues, including epidermal, cardiovascular, and cerebrovascular sites. Therefore, pharmacologic inhibition of CTSK causes an increased risk of off-target cerebrovascular accidents in clinical trials.^14^, ^15^, ^16^ Thus, we aimed to detarget the transduction of rAAV9 to non-skeletal tissues by utilizing a bone-homing capsid. Previously reported peptide motifs that can direct a liposome to osteoblast-enriched bone-forming surfaces, (Asp-Ser-Ser)_6_,^31^ or OC-enriched bone-resorbing surfaces, (Asp)_14_,^32^ were grafted onto the N terminus of the VP2 subunit of the AAV9 capsid protein^26^ (Figure 4A). Since hydroxyapatite (HA) is a major inorganic component in the bone tissue,^33^ we tested the capsid for HA-binding affinity *in vitro*. Genome copies (GC) of AAV9.DSS-Nter (rAAV9.DSS) and AAV9.D14-Nter (rAAV9.D14) in the HA pellet were markedly increased relative to those of rAAV9 (Figure 4B, left), while GC of these vectors in the supernatant were decreased (Figure 4B, right), demonstrating that both of the AAV9.DSS-Nter and AAV9.D14-Nter capsids can enhance HA-binding affinity. Of note, capsid grafting of these motifs did not affect rAAV9’s transduction efficiency in OCs (Figure 4C). To test the capsid for bone-targeting activity *in vivo*, vectors were i.v. injected into 2-month-old mice and vector biodistributions were assessed by EGFP expression using IVIS optical imaging 2 weeks post-injection (Figures 4D and 4E; Figure S6A). rAAV9.DSS-Nter-treated mice exhibited less EGFP expression than that achieved by rAAV9 in the liver (~50% less) and muscle (~30% less), and little to no expression in the heart. However, the biodistributions of rAAV9.D14-Nter were comparable to those of rAAV9, suggesting that the AAV9.D14-Nter capsid is unable to confer bone-homing specificity of rAAV9 *in vivo*. These results are consistent with fluorescence microscopy data showing a nearly complete lack of EGFP expression in the heart and a significant reduction in the liver and muscle of rAAV9.DSS-Nter-treated mice when compared with mice treated with rAAV9 or rAAV9.D14-Nter (Figure 4F; Figure S6B). Importantly, expression in femurs was relatively comparable between treatment groups (Figures 4D and 4F), demonstrating that the AAV9.DSS-Nter capsid, not the AAV9.D14-Nter capsid, reduces transduction from non-skeletal tissues. Likewise, GC of AAV9.DSS-Nter in the liver were markedly decreased relative to those of rAAV9, whereas GC of these vectors in the bone were relatively comparable (Figure S6C). We note that with the doses of vector used in these experiments (8 × 10^11^ GC/mouse), we did not anticipate strong transduction of brain tissues following i.v. administration (Figure 4F). Taken together, the DSS-VP2 capsid protein, not the D14-VP2 capsid protein, improves bone-homing specificity of rAAV9 by detargeting its transduction to non-relevant tissues.

### Bone-targeting rAAV9-Mediated Silencing of *ctsk* Counteracts Bone Loss in Mouse Models of Osteoporosis

Postmenopausal (type 1) and senile (type 2) osteoporosis results in severe bone loss and deterioration of bone structure, increasing the risk of fractures. Bone loss in postmenopausal women is caused by enhanced OC activity as a result of estrogen withdrawal. Estrogen, which is normally produced as a part of the menstrual cycle, mainly acts on OCs as a negative regulator, preventing OC-mediated bone resorption.^34^ Senile osteoporosis typically develops after the age of 70 for both men and women, and is a consequence of bone senescence and calcium deficiency. To test therapeutic effects of our bone-targeting rAAV9-mediated gene silencing in osteoporosis, we packaged *amiR-ctsk* with AAV9.DSS-Nter capsid. Compared to rAAV9.*amiR-ctsk*, the rAAV9.DSS-*amiR-ctsk* showed a modest reduction in infectivity at lower doses (5 × 10^10^ GC/kg) (Figure S7A), but effectively silenced expression of *ctsk* (Figure S7B) and inhibited OC resorption activity (Figure S7C) without any alteration in TRAP activity at a dose of 5 × 10^12^ GC/kg. These results demonstrate that there are no adverse effects of the AAV9.DSS-Nter capsid on the therapeutic potential of *amiR-ctsk*.

**Figure 5:**
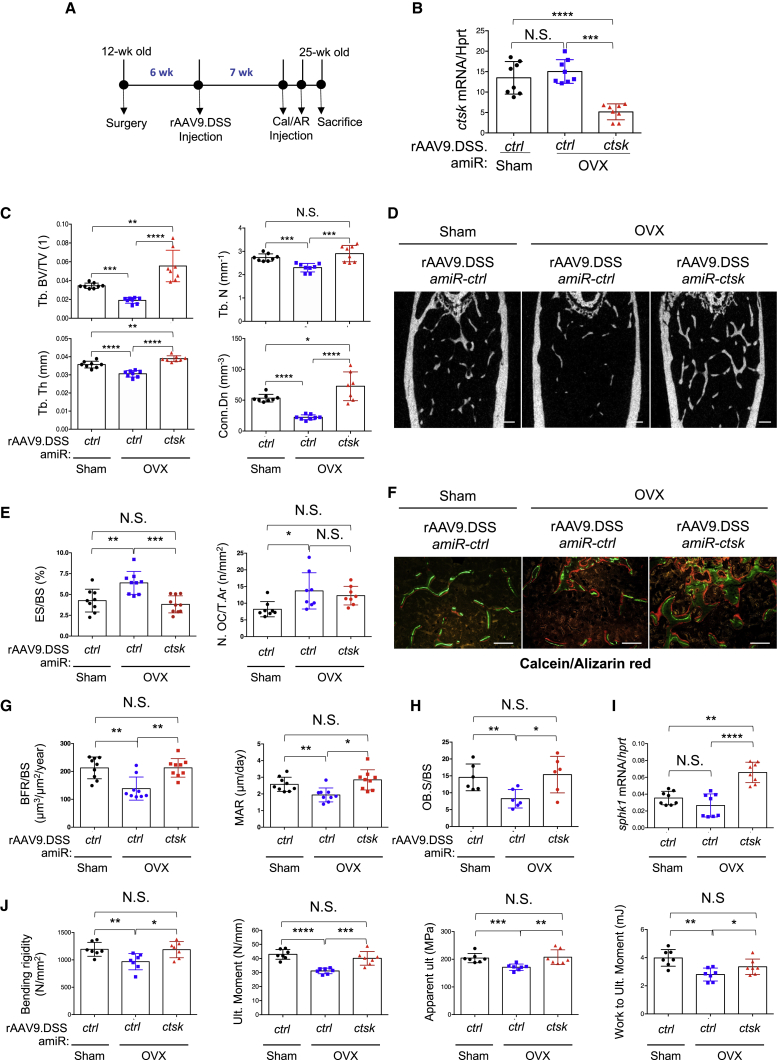
Bone-Targeting AAV9-Mediated Silencing of *ctsk* Reverses Bone Loss in a Mouse Model of Postmenopausal Osteoporosis (A) Timeline of the study and treatment methods. (B–J) Sham or OVX surgery was performed on 3-month-old female mice. Six weeks later, a single dose of 8 × 10^11^ GC of rAAV9.DSS-Nter carrying *amiR-ctrl* or *amiR-ctsk* was i.v. injected. (B) Seven weeks after injection, *ctsk* mRNA levels were assessed in the tibia (B, n = 8/group). Femoral trabecular bone mass was assessed by microCT. (C and D) Representative 3D reconstruction (D) and relative quantifications (C) are displayed (n = 8/group). (E–H) Histomorphometric quantification of ES/BS, N.OC/T.Ar (E), representative calcein/alizarin red labeling (F), BFR/BS, MAR (G), and OB.S/BS (H) are displayed (n = 8–9/group). (I) *sphk1* mRNA levels in the tibia were assessed by RT-PCR and normalized to *hprt* (n = 8). (J) Femoral biomechanical properties, including bending rigidity and moment, apparent bending stress, and work to bending moment were quantified (n = 7/group). Values represent mean ± SD. *p < 0.05, **p < 0.01, ***p < 0.001, ****p < 0.0001 by an unpaired two-tailed Student’s t test and one-way ANOVA test. N.S., not significant.

Ovariectomized (OVX) mice are an established model for postmenopausal osteoporosis induced by estrogen deficiency.^35^ Sham or OVX surgery was conducted on 3-month-old female mice and rAAV9.DSS-*amiR-ctsk* was i.v. injected 6 weeks post-surgery (Figure 5A). Seven weeks after injection, reduced levels of *ctsk* mRNAs were validated in *amiR-ctsk-*expressing OVX femurs (Figure 5B). While *amiR-ctrl*-expressing OVX mice showed a significant reduction in trabecular bone mass relative to sham mice, bone loss was completely reversed in the femurs of *amiR-ctsk*-expressing OVX mice, as demonstrated by the greater trabecular BV/total volume (TV), thickness, number, and connectivity density (Figures 5C and 5D). These femurs displayed a decrease in bone erosion surfaces (Figure 5E, left), while the number of OCs in the total bone area is relatively comparable to that of *amiR-ctrl*-expressing OVX femurs (Figure 5E, right). Additionally, treatment with rAAV9.DSS-*amiR-ctsk* in OVX mice enhances BFR, MAR, and Ob.S/BS (Figures 5F–5H), which is accompanied with elevated levels of s*phk1* mRNA (Figure 5I). This effect is specific to the bone tissue, as no mineralization was detected in non-skeletal tissues, including brain, heart, liver, and skeletal muscle (Figure S8). Finally, biomechanical testing analysis showed that the strength and stiffness of femurs were considerably protected from OVX-induced bone loss when treated with rAAV9.DSS-*amiR-ctsk* (Figure 5J), suggesting that bone-targeting rAAV9-mediated silencing of *ctsk* improves clinically meaningful endpoints in osteoporotic mice. Taken together, these results demonstrate that systemic delivery of *amiR-ctsk* by the bone-tropic capsid can counteract bone loss and enhance clinically relevant mechanical properties of bone after the onset of estrogen deficiency-induced osteoporosis by simultaneously suppressing OC-mediated bone resorption and promoting osteoblast-mediated bone formation.

**Figure 6:**
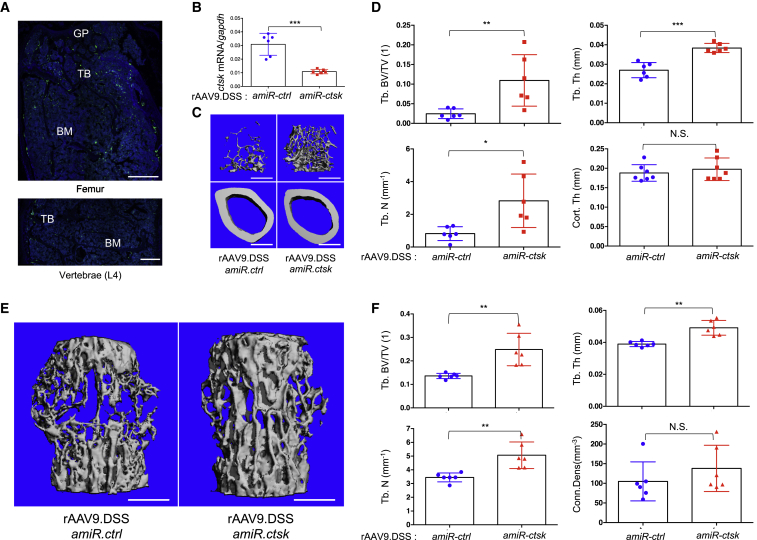
Bone-Targeting AAV9-Mediated Silencing of *ctsk* Prevents Bone Loss in a Mouse Model of Senile Osteoporosis A single dose of 8 × 10^11^ GC of rAAV9.DSS carrying *amiR-ctrl* or *amiR-ctsk* was i.v. injected into 18-month-old male mice. (A) Two months later, EGFP expression in cryosectioned femurs and lumbar vertebrae (L4) were assessed by fluorescence microscopy. (B) Levels of *ctsk* mRNA in the tibia were assessed by RT-PCR (n = 6/group). Trabecular bone mass and cortical thickness in femurs and lumbar vertebrae (L4) were assessed by microCT. (C–F) Representative 3D-reconstruction (C and E) and relative quantification (D and F) are displayed (n = 6/group). Scale bars, 500 μm. Values represent mean ± SD. ∗p < 0.05, ∗∗p < 0.01, ∗∗∗p < 0.001 by an unpaired two-tailed Student’s t test and one-way ANOVA test. N.S., not significant.

Next, we tested therapeutic effects of rAAV9.DSS-*amiR-ctsk* in a mouse model of senile osteoporosis. Vector was i.v. injected into 18-month-old male mice and 2 months later, EGFP expression in femurs and lumbar vertebrae was assessed by fluorescence microscopy (Figure 6A). EGFP-expressing cells are mainly located on the surface of trabecular bones in femurs and vertebrae. Compared to *amiR-ctrl-*treated animals, mice injected with *amiR-ctsk* showed reduced levels of *ctsk* mRNA (Figure 6B) and a relative increase in trabecular bone mass within the femur and lumbar vertebrae, as indicated by greater trabecular BV/TV, thickness, and number (Figures 6C–6F). As higher expression of EGFP is detected in femurs relative to vertebrae, bone accrual in femurs was greater than in vertebrae of mice treated with rAA9.DSS.*amiR-ctsk*. Thus, these results demonstrate that bone-targeted delivery of *amiR-ctsk* is also effective at reversing bone loss in senile osteoporosis. Collectively, rAAV9.DSS-*amiR-ctsk* is a promising therapeutic agent for both postmenopausal and senile osteoporosis.

## Discussion

Our previous study demonstrated that bone-targeting rAAV9-mediated silencing of Schnurri-3 in osteoblasts is a bone-anabolic therapy for osteoporosis that does not alter OC development. This study establishes a promising osteoporosis therapy that simultaneously suppresses OC-mediated bone resorption and promotes osteoblast-mediated bone formation, while limiting adverse off-target effects. In addition to osteoblasts, AAV9 targets OCs to drive RNAi-mediated silencing of the key OC regulators RANK and CTSK, which we show have clinical relevance as genetic targets to prevent osteoporosis. Since rAAV9 can transduce both osteoblasts and OCs *in vivo*,^26^ our OC-targeting rAAV9-based gene therapy can complement our previous approach, which only targeted osteoblasts for the treatment of osteoporosis.

Unlike other RNAi-based strategies using liposomes^31^ or lipid-based nanoparticles,^36^ a single systemic injection of rAAV9.DSS.*amiR-ctsk* is sufficient to increase bone accrual in mice. Due to their high-transduction efficiency, ability to confer persistent transgene expression, and lack of post-infection immunogenicity and pathogenicity, AAV vectors have a long track record for safety and efficacy in relevant preclinical and clinical studies.^37^ In particular, rAAV9-mediated gene therapy is currently the leading platform for the treatment of neurological disorders,^38^ as systemically delivered rAAV9 can traverse the blood-brain barrier to target the central nervous system (CNS).^39^ Additionally, our previous study and others have demonstrated its ability to transduce various peripheral tissues, such as liver, retina, striated muscles, and bone in adult mice.^26^^,^^40^^,^^41^ rAAV9 is likely to be effective for the transduction of osteoblasts and OCs that reside in the trabecular bone of femur with high bone remodeling activity relative to lumbar vertebrae. This is accompanied with greater bone accrual in femurs relative to vertebrae when treated with rAAV9.*amiR-ctsk*. Notably, treatment with rAAV9.*amiR-ctsk* results in impaired bone resorption activity and elevated expression of *sphk1*, which produces S1P to promote differentiation of neighboring RUNX2^+^ osteoblasts. Therefore, bone-targeting rAAV9-mediated silencing of *ctsk* in OCs is a potent gene therapy to treat osteoporosis, while limiting adverse off-target effects.

RANK and CTSK are promising therapeutic targets for osteoporosis. Denosumab, the human monoclonal IgG2 antibody against RANKL,^10^ and odanacatib, the pharmacologic CTSK inhibitor,^14^ are both effective in preventing bone loss in human patients with osteoporosis. However, continuous treatment with denosumab reduces physiologic bone remodeling and can potentially be accompanied by adverse effects.^3^ Furthermore, pharmacological inhibition of CTSK causes off-target cerebrovascular accidents.^16^ It was therefore apparent that the safety of rAAV9 in the brain be considered. Two bone-homing peptide motifs, (Asp-Ser-Ser)_6_^31^ and (Asp)_14_,^32^ have been developed to direct liposomes to osteoblast-enriched bone-forming and OC-enriched bone-resorbing surfaces, respectively. Therefore, display of the (Asp)_14_ peptide on the capsid protein may enhance rAAV9’s ability to transduce OCs *in vivo*. To address this, we rationally designed an AAV9 capsid by grafting the peptide motif (Asp)_14_ or (Asp-Ser-Ser)_6_ onto AA9-VP2. Although both AAV9.D14-Nter and AAV9.DSS-Nter capsids enhance *in vitro* HA-binding affinity, only the AAV9.DSS-Nter capsid can detarget transduction from non-skeletal tissues in mice, while retaining transduction in OCs and osteoblast-lineage cells^26^ that reside on the BS. This capability demonstrates its ability to specifically target both OC-enriched bone-resorbing areas and osteoblast-enriched bone-forming areas. We note that the transduction efficiencies of rAAVs *in vitro* are scarcely predictive of their *in vivo* performance. This phenomenon may be due to the presence of multiple physiological barriers related to the route of administration, serum factors, circulating neutralizing antibodies, and extracellular barriers.^42^ Future vector improvements to transduce exclusively OC lineage cells, such as using OC-specific promoters in the vector genome design or further engineering of capsids, will allow for even more precise bone-targeting rAAV vectors to be developed. Finally, the bone-targeted, rAAV9-mediated silencing of *ctsk* in OCs may have clinical utility for counteracting bone loss in other skeletal diseases, such as inflammatory arthritis-induced bone loss.

## Materials and Methods

### rAAV Vector Design and Production

The amiR against the mouse *ctsk* transcript was designed by using a custom Excel macro, which considers miR-33 scaffold design rules to generate optimized amiR cassettes.^28^ DNA sequences for *amiR-33-ctrl*, *amiR-33-rank-1* and -*2*, and *amiR-33-ctsk-1* and -*2* were synthesized as gBlocks and cloned into the intronic region of the pAAVsc-*CB6-EGFP* plasmid at PstI and BglII restriction enzyme sites.^43^ Constructs were verified by Sanger sequencing. Plasmids of the pAAV-*amiR-ctrl*, pAAV-*amiR-rank-1* and -*2*, and pAAV-*amiR-ctsk-1* and -*2* were packaged with AAV9 capsids. rAAV production was performed by transient transfection of HEK293 cells, purified by CsCl sedimentation, and titered by droplet digital PCR (ddPCR) on a QX200 ddPCR system (Bio-Rad) using the *EGFP* primer/probe set as previously described.^44^ The sequences of gBlocks and oligonucleotides for ddPCR and are listed in Table S1. The bone-targeting rAAV9 vectors (AAV9.D14-Nter and AAV9.DSS-Nter) were generated by inserting the codon-optimized DNA sequence encoding the bone-targeting peptide motif, D14 (Asp)_14_ or DSS (AspSerSer)_6_, into the AAV9 capsid protein VP2, as previously described.^45^

### Animals

C57BL/6J and BALB/cJ mice were purchased from Jackson Laboratory. For systemic delivery, 200 μL of rAAV9 carrying *amiR-ctrl*, *amiR-rank-1* and *-2*, or *amiR-ctsk-1* and *-2* (8 × 10^11^ GC/mouse) was i.v. injected into mice. Mouse models of postmenopausal osteoporosis were generated by anesthetizing and bilaterally ovariectomizing 3-month-old female mice (C57BL/6J). Six weeks after the surgery, sham or OVX mice were i.v. injected with 200 μL of rAAV9.DSS-Nter carrying *amiR-ctrl* or *amiR-ctsk* (8 × 10^11^ GC/mouse). Mice were randomly divided into three groups with sham + rAAV9.DSS-Nter-*amiR-ctrl*, OVX + rAAV9.DSS-Nter-*amiR-ctrl*, and OVX + rAAV9.DSS-Nter-*amiR-ctsk*. As a mouse model of senile osteoporosis, 18-month-old male mice were i.v. injected with 200 μL of rAAV9.DSS-Nter carrying *amiR-ctrl* or *amiR-ctsk* (8 × 10^11^ GC/mouse). All animals were used in accordance with the NIH *Guide for the Care and Use of Laboratory Animals* and were handled according to protocols approved by the University of Massachusetts Medical School Institutional Animal Care and Use Committee (IACUC) on animal care.

### MicroCT Analysis

Micro-computed tomography (microCT) was used for qualitative and quantitative assessment of trabecular and cortical bone microarchitecture. Analysis was carried out by an investigator blinded to the genotypes of the animals. Femurs excised from the indicated mice were fixed with 10% neutral buffered formalin and scanned using a microCT 35 (Scanco Medical) with a spatial resolution of 7 μm. For trabecular bone analysis of the distal femur, an upper 2.1-mm region beginning 280 μm proximal to the growth plate was contoured. Connectivity density (Conn.D) was calculated with the Conn-Euler method of Odgaard and Gundersen^46^ using the Scanco microCT 35 software. Based on a 3D node-and-branch network of a cancellous bone structure (connectivity), its connectivity density was calculated by dividing the connectivity estimate by the volume of the sample. For cortical bone analyses of femurs, midshaft regions of 0.6 mm in length were used. 3D reconstruction images were obtained from contoured 2D images by methods based on distance transformation of the binarized images. Alternatively, the Inveon multimodality 3D visualization program was used to generate fused 3D viewing of multiple static or dynamic volumes of microCT modalities (Siemens Medical Solutions USA). All images presented are representative of the respective genotypes (n > 6).

### Histology, Histomorphometry, and Immunofluorescence

For histological analyses, femurs and tibias were dissected from rAAV-treated mice, fixed in 10% neutral buffered formalin for 2 days, and decalcified by 5% tetrasodium EDTA for 2–4 weeks. Tissues were dehydrated by passage through an ethanol series, cleared twice in xylene, embedded in paraffin, and sectioned at a thickness of 6 μm along the coronal plate from anterior to posterior. Decalcified femoral sections were stained with TRAP.

For dynamic histomorphometric analysis, 25 mg/kg calcein (Sigma, C0875) and 50 mg/kg alizarin-3-methyliminodiacetic acid (Sigma, A3882) dissolved in 2% sodium bicarbonate solution were subcutaneously injected into mice in 6-day intervals. MARs and mineralized surface/BS to calculate BFRs were assessed by measuring the distances between BSs labeled by calcein (existing bone) and alizarin-3-methyliminodiacetic acid (newly formed bone). After fixation in 10% neutral buffered formalin for 2 days, undecalcified femur samples were embedded in methyl methacrylate, and the proximal metaphyses of femurs were sectioned longitudinally (5 μm) and stained with McNeal’s trichrome for osteoid assessment, toluidine blue for osteoblasts, and TRAP for OCs.^47^ A region of interest was defined in the trabecular bone of the metaphysis, and BFR/BS, MAR, BS, Ob.S/BS, and OC surface (Oc.S/BS) were measured using a Nikon Optiphot 2 microscope interfaced with a semiautomatic analysis system (OsteoMetrics). Measurements were taken on two sections/sample (separated by ~25 μm) and summed prior to normalization to obtain a single measure/sample in accordance with the American Society of Bone and Mineral Research (ASBMR) Histomorphometry Nomenclature Committee.^48^ This methodology has undergone extensive quality control and validation, and the results were assessed by two different researchers in a blinded fashion.

For immunofluorescence imaging, fresh femurs dissected from rAAV-treated mice were collected and immediately fixed in ice-cold 4% paraformaldehyde solution for 2 days. Semi-decalcification was carried out for 5 days in 0.5 M EDTA (pH 7.4) at 4°C with constant shaking, and infiltration was followed with a mixture of 20% sucrose in phosphate buffer for 1 day and 25% sucrose in phosphate buffer the next day. All samples were embedded in a 50/50 mixture of 25% sucrose solution and OCT compound (Sakura) and cut into 12-μm-thick sagittal sections using a cryostat (Leica). Immunofluorescence staining and analysis were performed as described previously.^47^^,^^49^ Briefly, after treatment with 0.2% Triton X-100 for 10 min, sections were blocked with 5% donkey serum at room temperature for 30 min and incubated overnight at 4°C with anti-CTSK antibody (A1782, ABclonal, 1:150). Primary antibodies were visualized with donkey anti-rat IgG Alexa Fluor 594 (1:500, Molecular Probes). Nuclei were counterstained with DAPI. An Olympus IX81 confocal microscope or Leica TCS SP5 II Zeiss LSM-880 confocal microscope was used to image samples.

### OC and Osteoblast Differentiation Analysis

For OC differentiation, bone marrow cells were flushed from the femurs and tibias of 2-month-old mice (C57BL/6J) and cultured in Petri dishes in α-minimal essential medium (α-MEM) medium with 10% fetal bovine serum (FBS) and 20 ng/mL M-CSF (R&D Systems) to obtain BMMs. 12 h later, nonadherent cells were re-plated into tissue culture dishes and cultured in the same medium for 2 days. BMMs were then differentiated into OCs in the presence of RANKL (20 ng/mL; R&D Systems) and M-CSF (20 ng/mL; R&D Systems) for 6 days. Alternatively, cells were plated into Osteo Assay surface plates (3987, Corning Life Sciences), and OC resorption activity was measured according to the manufacturer’s instructions.

For osteoblast differentiation, calvarial osteoblasts (COBs) were isolated from 4-day-old mice by enzymatic digestion in α-MEM containing 0.5 mg/mL collagenase-P (Roche) and 0.05% trypsin and cultured in osteogenic medium (0.1 mg/mL ascorbic acid, 10 mM β-glycerophosphate). COBs were incubated with 10-fold diluted Alamar Blue solution (Invitrogen, DAL1100) for cell proliferation. Subsequently, cells were washed and incubated with a solution containing 6.5 mM Na_2_CO_3_, 18.5 mM NaHCO_3_, 2 mM MgCl_2_, and phosphatase substrate (Sigma, S0942), and alkaline phosphatase activity was measured by an iMark microplate absorbance reader (Bio-Rad). To assess extracellular matrix mineralization in mature osteoblasts, cells were washed twice with phosphate-buffered saline (PBS) and fixed in 70% EtOH for 15 min at room temperature. Fixed cells were washed twice with distilled water and then stained with a 2% alizarin red solution (Sigma, A5533) for 5 min. Cells were then washed three times with distilled water and examined for the presence of calcium deposits.

### Flow Cytometry

Bone marrow cells were flushed from the femurs and tibias of 2-month-old mice (C57BL/6J) and incubated for 2 min at room temperature with BD Pharm Lyse hypotonic lysis buffer (BioLegend, 420301) for red blood cell (RBC) lysis. Cells were washed twice with cold fluorescence-activated cell sorting (FACS) buffer, incubated with Fc blocking buffer (BD Biosciences, 564765) for 15 min at 4°C, and treated with antibody cocktail including CD11b (Tonbo Biosciences, 20-0112), CD45R/B220 (Tonbo Biosciences, 65-0452), CD117 (Tonbo Biosciences, 60-1172), CD3 (Tonbo Biosciences, 50-0031), Ter119 (BioLegend, 116233), and Ly6C (BioLegend, 128017) in cold FACS buffer. After treatment with DAPI, cells were subjected to FACS analysis using a BD LSR II flow cytometer (BD Biosciences). The data were analyzed using FlowJo (v.10.1).

### HA-Binding Assay

HA beads (1 mg/mL) were suspended in 25 mM Tris-HCl (pH 7.4). Three different GC (10^8^, 10^9^, 10^10^) of rAAV9, rAAV9.D14-Nter, and rAAV9.DSS-Nter were incubated with HA in a 100-μL vol for 1 h at 37°C and 300 rpm, and then vector titers in the HA pellet and the supernatant were measured by ddPCR.^44^

### ELISA Analysis

CTX1 ELISA (EM0960, FineTest) analysis was performed according to the manufacturer’s instructions.

### Quantitative RT-PCR Analysis

Total RNA was purified from cells using QIAzol (QIAGEN), and cDNA was synthesized using a high-capacity cDNA reverse transcription kit from Applied Biosystems. Quantitative RT-PCR was performed using SYBR Green PCR master mix (Bio-Rad) with a CFX Connect RT-PCR detection system (Bio-Rad). To measure mRNA levels in bone tissues, tibias were removed of their bone marrow and snap-frozen in liquid nitrogen for 30 s and homogenized in 1 mL of QIAzol for 1 min. Primers used for PCR are described in Table S1.

### Statistical Analysis

Except where indicated, all data are graphically represented as the mean ± SD. For experiments with three or more samples, statistical analysis was performed using one-way ANOVA followed by a Bonferroni-corrected Student’s t test. For two-sample comparisons, a two-tailed, unpaired Student’s t test was applied. Values were considered statistically significant at p <0.05. Results shown are representative of three or more individual experiments.

## Author Contributions

Y.-S.Y. designed, executed, and interpreted the experiments. J.X. designed and generated all of the AAVs used in this work. D.W. developed the AAV9.DSS-Nter and AAV9.D14-Nter capsids. J.-M.K. performed ovariectomy. S.C., J.-H.K., and S.-K.S. performed flow cytometry, OC culture, histology, and histomorphometry. E.G. and P.W.L.T interpreted the experiments and helped draft the manuscript. G.G. and J.-H.S. supervised the research and prepared the manuscript.

## Conflicts of Interest

G.G. and J.-H.S. have submitted a patent application concerning the methodology described in this study. G.G. and J.H.-S. are scientific co-founders of AAVAA Therapeutics and hold equity in this company. G.G. is also a scientific co-founder of Voyager Therapeutics and Aspa Therapeutics and holds equity in these companies. G.G. is an inventor on patents with potential royalties licensed to Voyager Therapeutics, Aspa Therapeutics, and other biopharmaceutical companies.

## Supplemental Information

Document S1. Figures S1–S8 and Table S1

Document S2. Article plus Supplemental Information
