# Supplementary material for: Bone-Targeting AAV-Mediated Gene Silencing in Osteoclasts for Osteoporosis Therapy
Source: Mol Ther Methods Clin Dev. 2020 Apr 18;17:922–35. doi: 10.1016/j.omtm.2020.04.010 (PMC7210389; doi:10.1016/j.omtm.2020.04.010)
Supplement: Document S1. Figures S1–S8 and Table S1 [file mmc1.pdf]

OMTM, Volume *17*

Supplemental Information

Bone-Targeting AAV-Mediated Gene Silencing in Osteoclasts for Osteoporosis Therapy

Yeon-Suk Yang, Jun Xie, Sachin Chaugule, Dan Wang, Jung-Min Kim, JiHea Kim, Phillip W.L. Tai, Seok-kyo Seo, Ellen Gravallese, Guangping Gao, and Jae-Hyuck Shim

Supplementary Information

Bone-targeting AAV9-mediated gene silencing in osteoclasts for osteoporosis therapy

Yeon-Suk Yang[1], Jun Xie[2, 3, 4], Sachin Chaugule[1], Dan Wang[2, 3], Jung-Min Kim[1], JiHea Kim[1], Phillip W.L. Tai[2, 3], Seok-kyo Seo[5], Ellen Gravallese[6], Guangping Gao[2, 3, 4, 7]*, and Jae-Hyuck Shim[1,7]*

[1]Division of Rheumatology, University of Massachusetts Medical School, Worcester, MA, USA
[2]Horae Gene Therapy Center, [3]Department of Microbiology and Physiological Systems, [4]Viral Vector Core, University of Massachusetts Medical School, Worcester, MA, USA

[5]Department of Obstetrics and Gynecology, Severance Hospital, Yonsei University College of Medicine, Seoul, Korea
[6]Division of Rheumatology, Immunology and Allergy, Brigham and Women's Hospital, Boston, MA, USA
[7]Li Weibo Institute for Rare Diseases Research, University of Massachusetts Medical School, Worcester, MA, USA

*To whom correspondence should be addressed.

Jae-Hyuck Shim and Guangping Gao

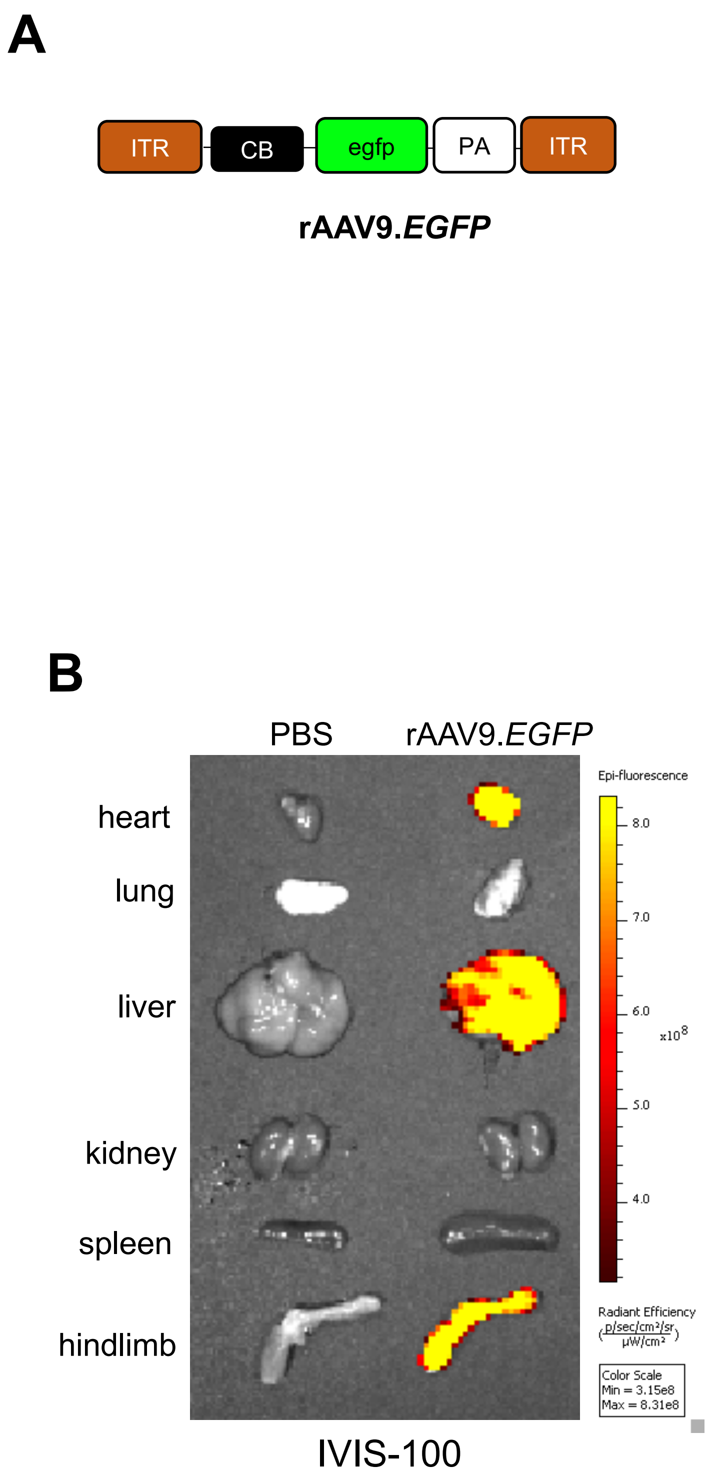

Supplementary Figure 1

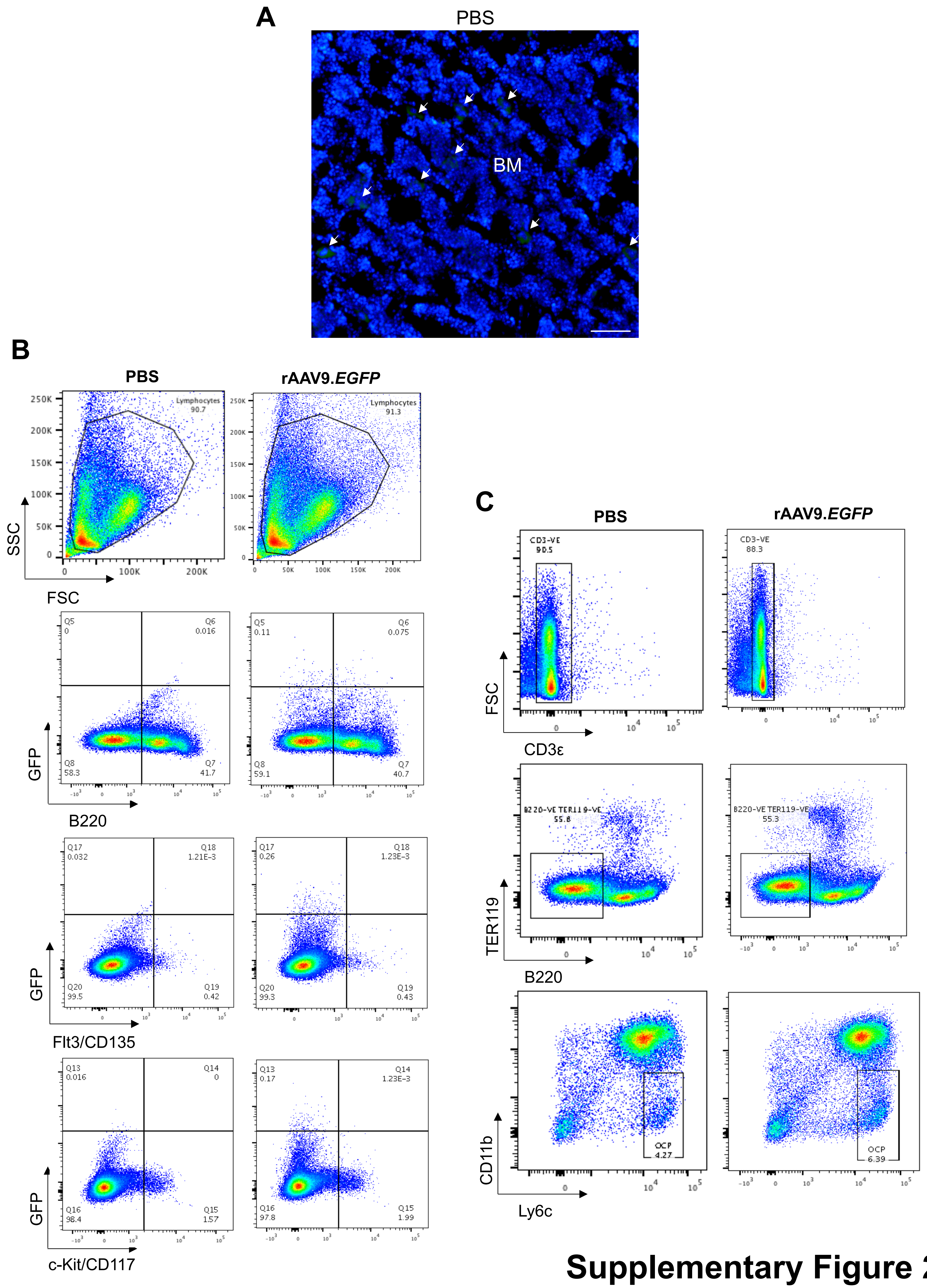

Supplementary Figure 2

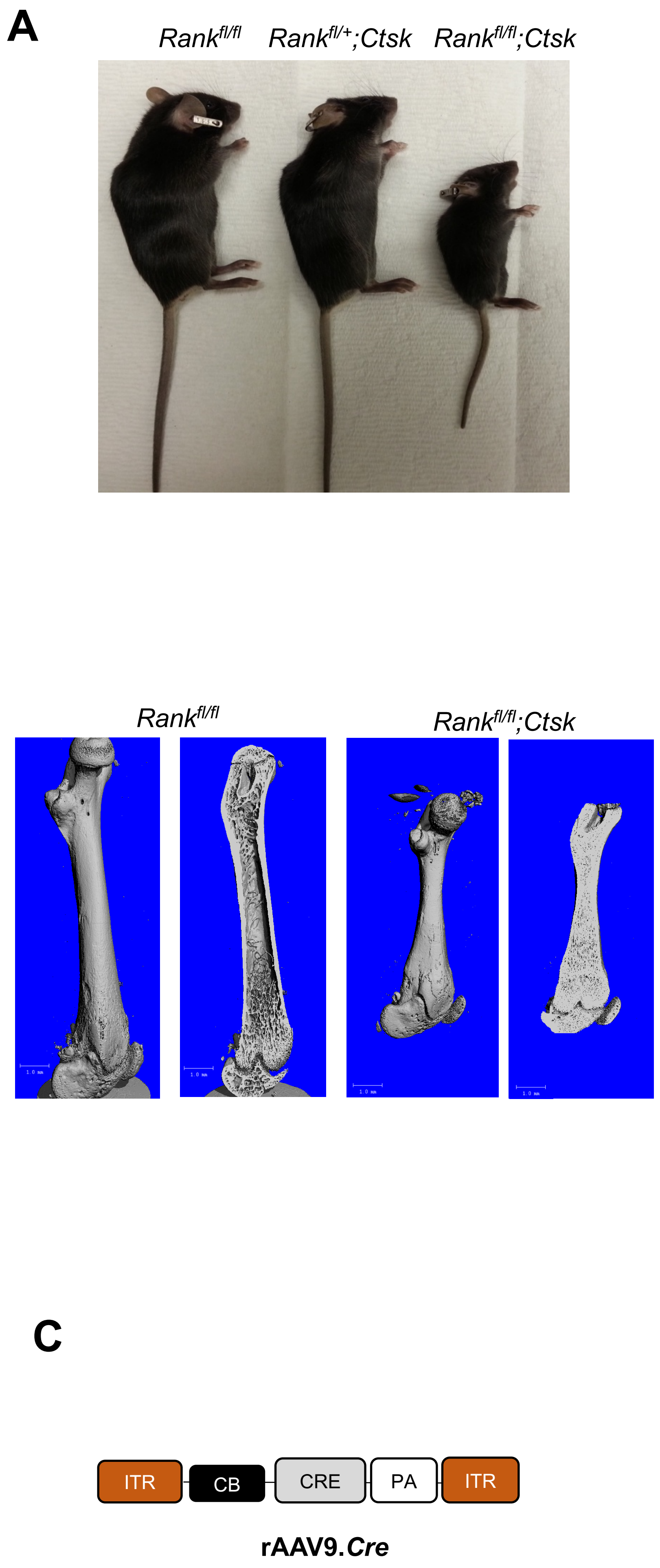

Supplementary Figure 3

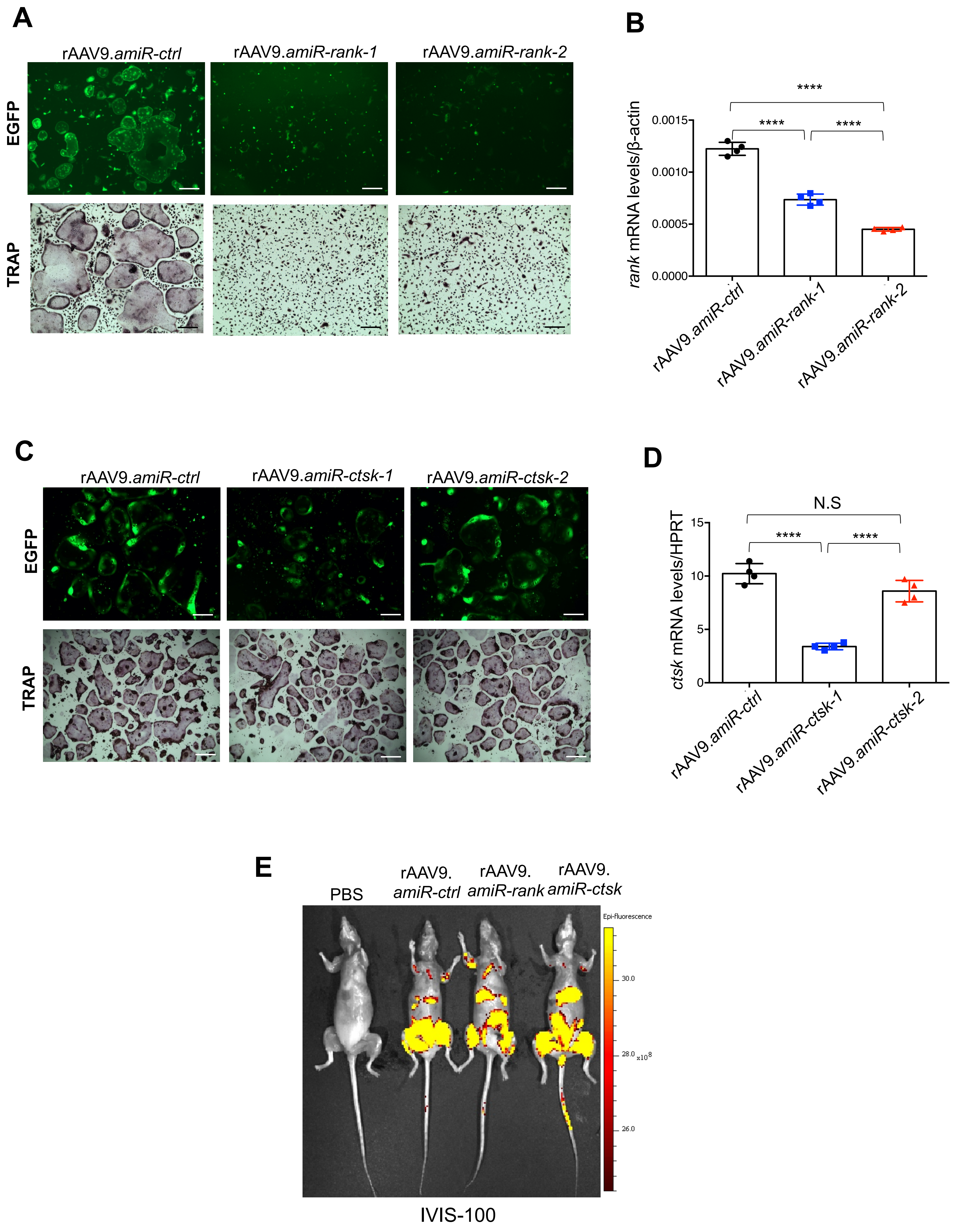

Supplementary Figure 4

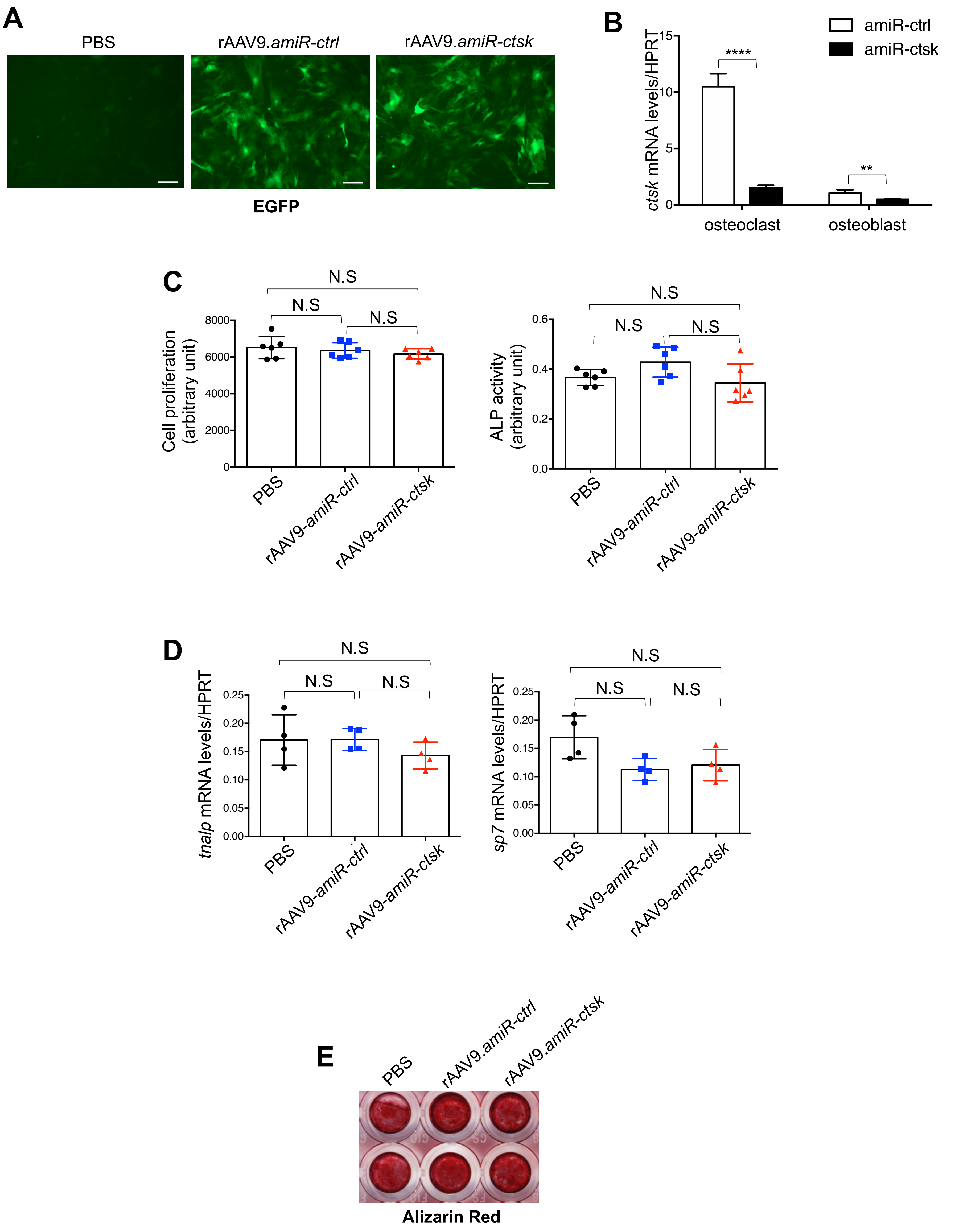

Supplementary Figure 5

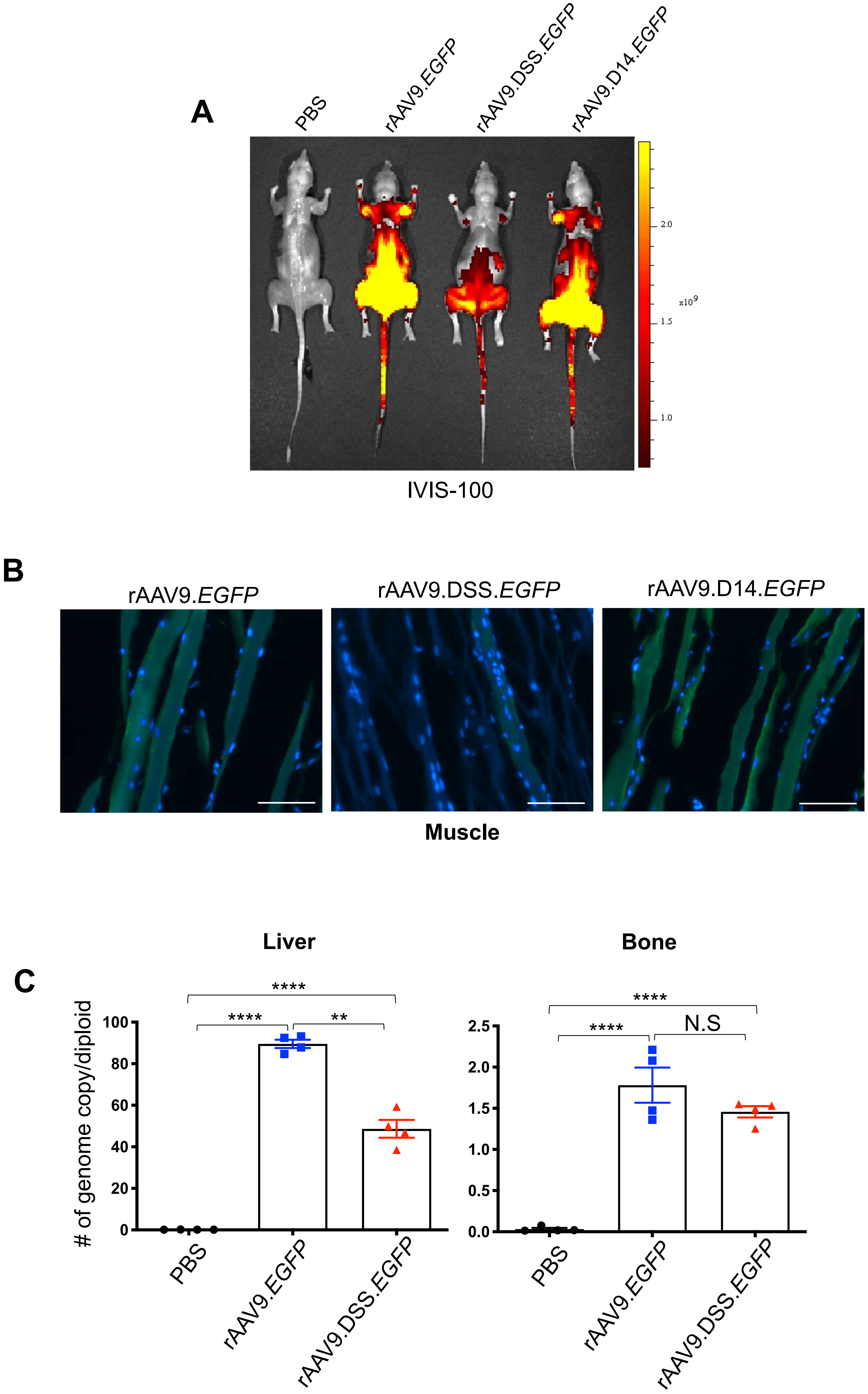

Supplementary Figure 6

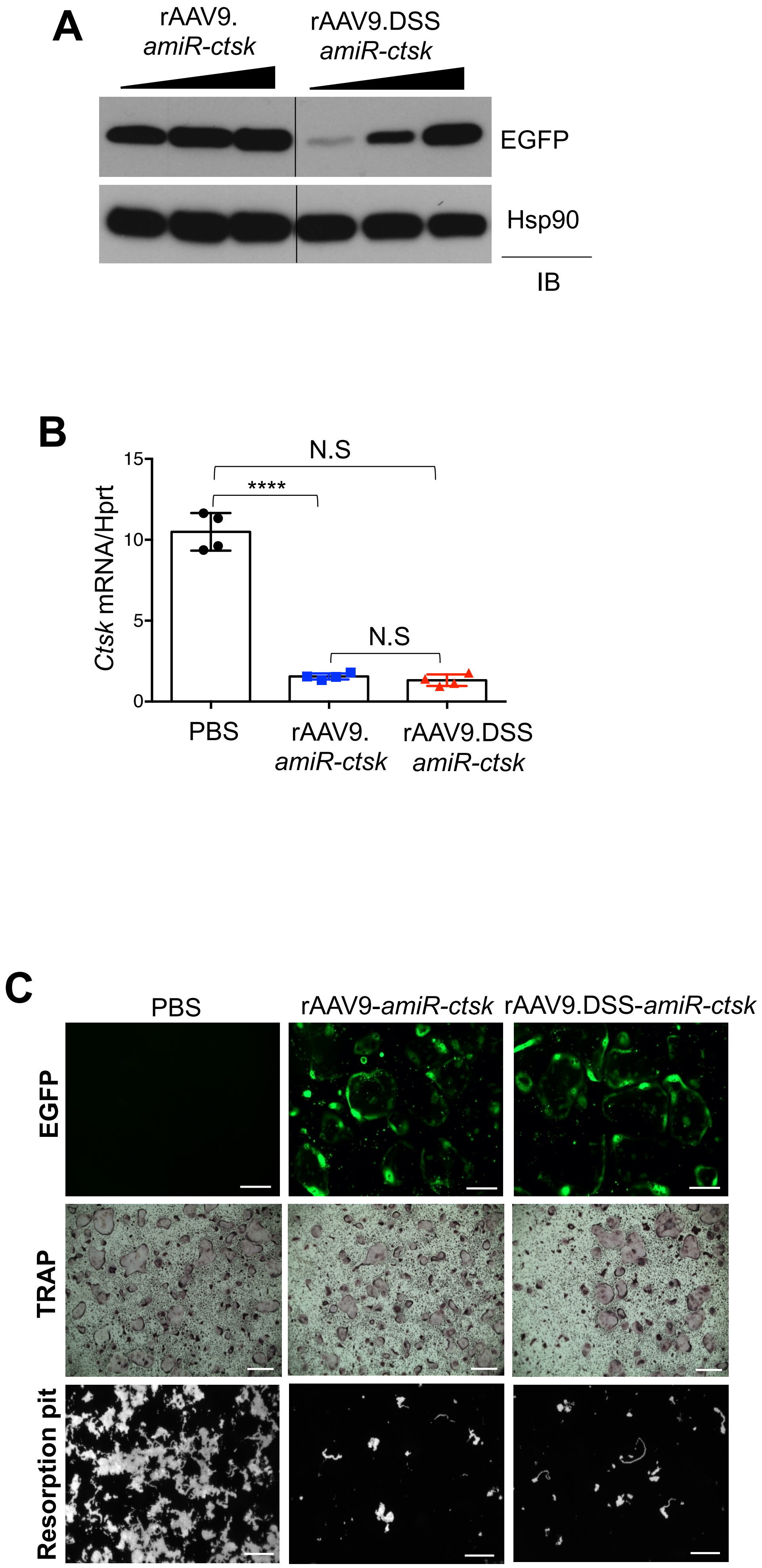

Supplementary Figure 7

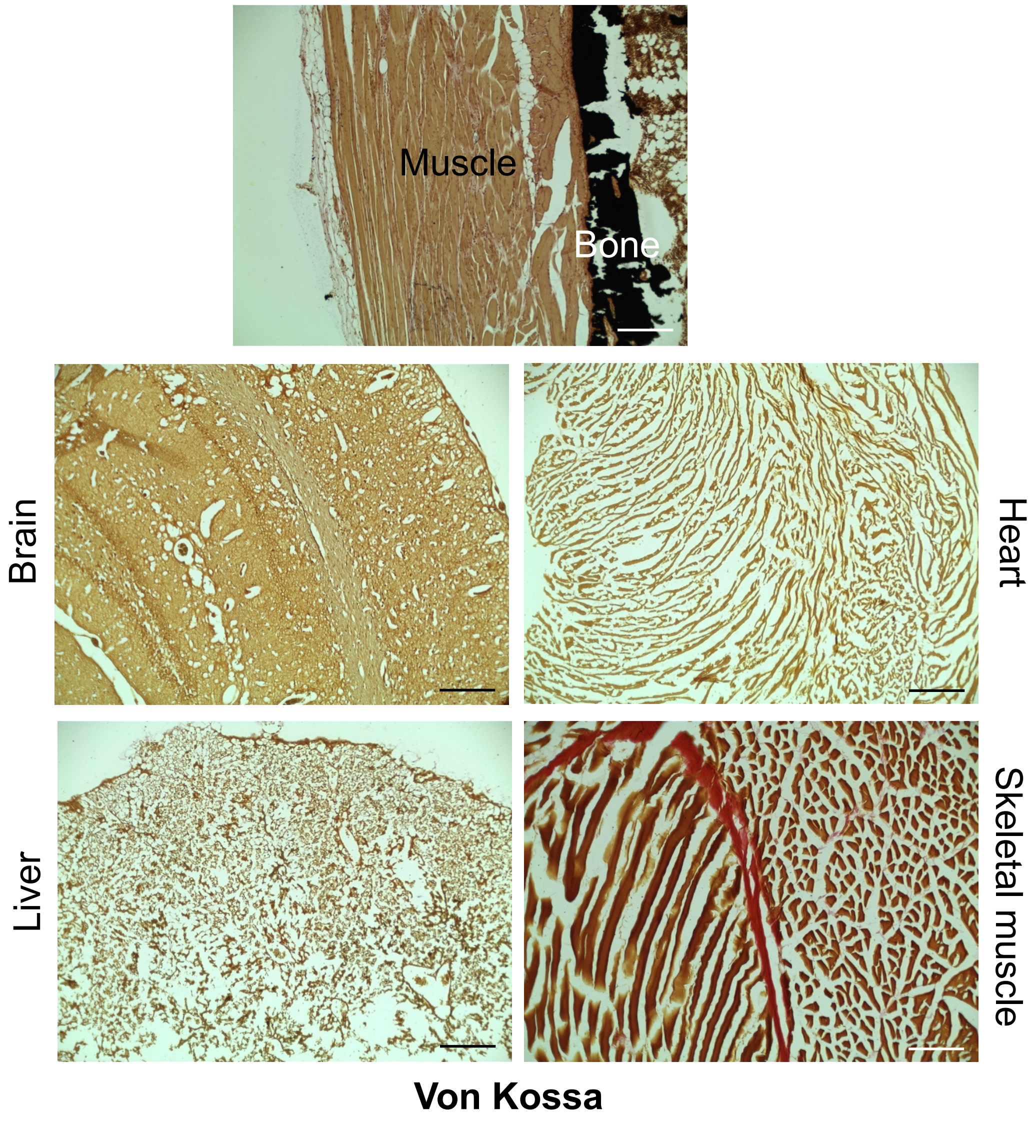

Supplementary Figure 8

Supplementary Figure legends

Supplementary Figure 1: Tissue distribution of systemically delivered rAAV9.*EGFP* in mice.

(A) Diagram of the rAAV9 construct containing a CMV enhancer/chicken β-actin promoter (*CB*), an *EGFP* reporter gene (*EGFP*), *β-globin* polyA sequence (PA), and inverted terminal repeats (ITR). **(B)** A single dose of PBS or 8 x 10^{11} genome copies (GCs) of rAAV.*EGFP* was intravenously (i.v.) injected into two-month-old male mice, and EGFP expression in individual tissues was quantified by IVIS-100 optical imaging two weeks post-injection. Scale bar represents relative fluorescence (p/sec/cm^2/sr/μW/cm^2).

Supplementary Figure 2: Identification of a subset of bone marrow cells transduced by systemically delivered rAAV9.

A single dose of PBS or 8 x 10^{11} genome copies (GCs) of rAAV.*EGFP* was intravenously (i.v.) injected into two-month-old male mice, and EGFP expression was assessed in cryo-sectioned femurs by fluorescence microscopy two weeks post-injection. Arrows indicate megakaryocytes with auto-fluorescence **(A)**. Cell-type specific evaluation of EGFP expression by isolation of bone marrow cells and staining with the indicated antibodies for quantification by flow cytometry **(B, C)**. Representative flow cytometry dot plots show the gating strategy of osteoclast progenitors (OCP; CD3ε^-, $B220^-$, $TER119^-$, $CD11b^{-/lo}$, $Ly6c^+$) described in **Figure 1C (C)**.

Supplementary Figure 3: Characterization of mice lacking Rank in cathepsin K-expressing osteoclasts

Mice with conditional alleles of *Rank* were crossed with transgenic mice with the cathepsin K promoter-driven expression of Cre recombinase. Representative image showing one-month-old female $Rank^{fl/fl}$ (wildtype), $Rank^{fl/+}$*;Ctsk* (heterozygote), and $Rank^{fl/fl}$*;Ctsk* (knockout) mice **(A**). MicroCT analysis of one-month-old $Rank^{fl/fl}$ and $Rank^{fl/fl}$*;Ctsk* femurs, demonstrating that deletion of *Rank* in osteoclasts results in osteopetrosis in mice **(B)**. Diagram of rAAV9 constructs containing the CMV enhancer/chicken β-actin promoter (CB), Cre recombinase (Cre), *β-globin* polyA sequence (PA), and inverted terminal repeats (ITR) **(C)**.

Supplementary Figure 4: Effects of rAAV9 carrying *amiR-rank* or *amiR-ctsk* on osteoclast differentiation and resorption activity *in vitro*.

(A-D) The amiR cassettes targeting two different positions of *rank* (*amiR-rank-1, -2*) or *ctsk* (*amiR-ctsk-1, -2*) mRNA were packaged with AAV9 capsids. Targeting sequences are described in **Table S1**. Bone marrow-derived monocytes (BMMs) harvested from two-month-old wildtype mice were treated with M-CSF and RANKL for two days to differentiate them into pre-osteoclasts (pre-OCs). Wildtype pre-OCs were transduced with either rAAV9 carrying *amiR-ctrl, amiR-rank-1, -2,* or *amiR-ctsk-1, -2* (10^{11} GC), and then cultured with M-CSF and RANKL for four days to differentiate them into mature osteoclasts. Transduction efficiency and osteoclast differentiation were assessed by EGFP expression and TRAP staining, respectively **(A, C)**. Levels of *rank* or *ctsk* mRNA were measured by RT-PCR and normalized to *hprt* **(B, D)** (n = 4/group). Scale bars: 1 mm. Values represent mean ± SD: N.S, not significant and ****, P < 0.0001 by an unpaired two-tailed Student's t-test and one-way ANOVA test.

(E) A single dose of PBS or 8 x 10^{11} GCs of rAAV9 carrying *amiR-ctrl, amiR-rank-2,* or *amiR-ctsk-1* was i.v. injected into two-month-old female mice, and two months later, EGFP expression

in whole body was monitored by IVIS-100 optical imaging. Scale bar represents relative fluorescence (p/sec/cm^2/sr/μW/cm^2).

Supplementary Figure 5: Effects of rAAV9.*amiR-ctsk* on osteoblast differentiation *in vitro*.

Two days after treatment with PBS, rAAV9.*amiR-ctrl,* or rAAV9.*amiR-ctsk*, primary calvarial osteoclast (COBs) were cultured under osteogenic conditions for six days and transduction efficiency was assessed by EGFP expression using fluorescence microscopy **(A)**. mRNA levels of *ctsk* **(B)** and osteogenic genes **(D)** were measured by RT-PCR and normalized to *hprt*, demonstrating that *ctsk* mRNA levels in COBs are significantly lower than those in mature osteoclasts derived from BMMs **(B)**. Alamar blue staining and alkaline phosphatase activity are displayed **(C)**. After 21 days of culturing, mineralization was assessed by alizarin red staining **(E)**. Scale bars: 1 mm. Values represent mean ± SD: N.S, not significant; **, $P < 0.01$; and ****, $P < 0.0001$ by an unpaired two-tailed Student's t-test and one-way ANOVA test.

Supplementary Figure 6: Tissue distribution of systemically-delivered rAAV9.DSS.*EGFP* in mice.

(A-C) A single dose of PBS or 8 x 10^{11} GCs of rAAV9, rAAV9.DSS-Nter, or rAAV9.D14-Nter was i.v. injected into two-month-old male mice. EGFP expression of the whole body was quantified by IVIS-100 optical imaging two weeks post-injection. Scale bar represents relative fluorescence (p/sec/cm^2/sr/μW/cm^2) **(A)**. Representative fluorescence microscopy images of cryo-sectioned skeletal muscle at high magnification. Scale bars: 50 μm **(B).** Genome copies of

PBS, rAAV9.*EGFP*, and rAAV9.DSS-Nter.*EGFP* in the liver and tibia were measured by ddPCR **(C)**.

Supplementary Figure 7: Effects of rAAV9.DSS-*amiR-ctsk* on osteoclast differentiation and resorption activity *in vitro.*

Two days after treatment with M-CSF and RANKL, wildtype pre-OCs were transduced with either rAAV9.*amiR-ctsk* or rAAV9.DSS-*amiR-ctsk* at three different multiplicities of infection (10^9, 10^{10}, 10^{11} GC), and cultured in the presence of M-CSF and RANKL for four days. EGFP expression was assessed by immunoblotting with anti-EGFP antibody **(A)** and fluorescence microscopy **(**10^{11} GC, **C)**. Hsp90 was used as a loading control. *ctsk* mRNA levels were measured by RT-PCR and normalized to *hprt* **(**10^{11} GC, **B)**. Osteoclast differentiation and resorption activity were assessed by TRAP activity and resorption pit assay, respectively **(**10^{11} GC, **D)**. Scale bars: 1 mm. Values represent mean ± SD: N.S, non-significant; ****, $P < 0.0001$ by an unpaired two-tailed Student's t-test and one-way ANOVA test.

Supplementary Figure 8: Effects of rAAV9.DSS-*amiR-ctsk* on non-skeletal tissues*.*

A single dose of 8 x 10^{11} GCs of rAAV9.DSS-*amiR-ctsk* was i.v. injected into two-month-old male mice. Two months later, Von Kossa staining was performed to assess abnormal calcification in cryo-sectioned femur, brain, heart, liver, and skeletal muscle. Scale bars: 100 μm**.**

Supplementary Table 1: Sequences of primers, probes and gblocks

Gene	Forward	Reverse
Mouse *Tnalp*	CACAATATCAAGGATATCGACGTGA	ACATCAGTTCTGTTCTTCGGGTACA
Mouse *Runx2*	TACAAACCATACCCAGTCCCTGTTT	AGTGCTCTAACCACAGTCCATGCA
Mouse *Sp7*	ATGGCGTCCTCTCTGCTTGA	GAAGGGTGGGTAGTCATTTG
Mouse *Sphnk1*	TGAGGTGGTGAATGGGCTAATGGA	AACAGCAGTGTGCAGTTGATGAGC
Mouse *Ctsk*	AGCAGAACGGAGGCATTGACTC	CCCTCTGCATTTAGCTGCCTTTG
Mouse *Hprt*	CTGGTGAAAAGGACCTCTCGAAG	CCAGTTTCACTAATGACACAAACG
EGFP	AGCAAAGACCCCAACGAGAA	GGCGGCGGTCACGAA
EGFP-probe	6FAM-CGCGATCACATGGTCCTGCTGG-TAMRA	
(AspSerSer)$_6$	GATTCATCAGATTCTTCTGATTCATCCGACTCTTCTGACAGTTCAGACAGCTCT	
(Asp)$_{14}$	GATGATGATGATGATGATGATGATGATGATGATGATGATGAT	
amiR-33-ctrl (*amiR-ctrl*)	tttgtcttttatttcaggtcccAGATCTAGGGCTCTGCGTTTGCTCCAGGTAGTCCGCTGCTCCCTT<br>GGGCCTGGGCCCACTGACAGCCCTGGTGCCTCTGGCCGGCTGCACACCTCCTGGCG<br>GGCAGCTGTGTTACTGTAGGATCGAGAGGGATGTTCTGGCAATACCTGTCCCTCTCC<br>TTACTACAGTAACACGGAGGCCTGCCCTGACTGCCCACGGTGCCGTGGCCAAAGAG<br>GATCTAAGGGCACCGCTGAGGGCCTACCTAACCATCGTGGGGAATAAGGACAGTGTC<br>ACCCCTGCAGgggatccggtggtggtgcaaatca	
amiR-33-rank1 (*amiR-rank-1*)	AGGGCTCTGCGTTTGCTCCAGGTAGTCCGCTGCTCCCTTGGGCCTGGGCCCACTGAC<br>AGCCCTGGTGCCTCTGGCCGGCTGCACACCTCCTGGCGGGCAGCTGTGAATGGTCC<br>ACATTTCAGGGACTGTTCTGGCAATACCTGGTCCCTGATTTATGGACCATTCACGGAG<br>GCCTGCCCTGACTGCCCACGGTGCCGTGGCCAAAGAGGATCTAAGGGCACCGCTGA<br>GGGCCTACCTAACCATCGTGGGGAATAAGGACAGTGTCACCC	
amiR-33-rank2 (*amiR-rank-2*)	AGGGCTCTGCGTTTGCTCCAGGTAGTCCGCTGCTCCCTTGGGCCTGGGCCCACTGAC<br>AGCCCTGGTGCCTCTGGCCGGCTGCACACCTCCTGGCGGGCAGCTGTGACAAATTA<br>GCTGTCAGCGCTGTTCTGGCAATACCTGGCGCTGACTGCAAATTTGTCACGGAGGCC<br>TGCCCTGACTGCCCACGGTGCCGTGGCCAAAGAGGATCTAAGGGCACCGCTGAGGG<br>CCTACCTAACCATCGTGGGGAATAAGGACAGTGTCACCC	
amiR-33-ctsk1 (*amiR-ctsk-1*)	tttgtcttttatttcaggtcccAGATCTAGGGCTCTGCGTTTGCTCCAGGTAGTCCGCTGCTCCCTT<br>GGGCCTGGGCCCACTGACAGCCCTGGTGCCTCTGGCCGGCTGCACACCTCCTGGCG<br>GGCAGCTGTGTTTCATCATAGTACACACCTCTGTTCTGGCAATACCTGGAGGTGTGAT<br>CCATGATGAAACACGGAGGCCTGCCCTGACTGCCCACGGTGCCGTGGCCAAAGAGG<br>ATCTAAGGGCACCGCTGAGGGCCTACCTAACCATCGTGGGGAATAAGGACAGTGTCA<br>CCCCTGCAGgggatccggtggtggtgcaaatca	
amiR-33-ctsk2 (*amiR-ctsk-2*)	tttgtcttttatttcaggtcccAGATCTAGGGCTCTGCGTTTGCTCCAGGTAGTCCGCTGCTCCCTT<br>GGGCCTGGGCCCACTGACAGCCCTGGTGCCTCTGGCCGGCTGCACACCTCCTGGCG<br>GGCAGCTGTGTTACTGTAGGATCGAGAGGGATGTTCTGGCAATACCTGTCCCTCTCC<br>TTACTACAGTAACACGGAGGCCTGCCCTGACTGCCCACGGTGCCGTGGCCAAAGAG<br>GATCTAAGGGCACCGCTGAGGGCCTACCTAACCATCGTGGGGAATAAGG	
